# Acknowledgment to the Reviewers of *Healthcare* in 2022

**DOI:** 10.3390/healthcare11030377

**Published:** 2023-01-29

**Authors:** Healthcare Editorial Office

**Affiliations:** MDPI AG, St. Alban-Anlage 66, 4052 Basel, Switzerland

High-quality academic publishing is built on rigorous peer review. *Healthcare* was able to uphold its high standards for published papers due to the outstanding efforts of our reviewers. Thanks to the efforts of our reviewers in 2022, the median time to first decision was 20 days and the median time to publication was 41 days. Regardless of whether the articles they examined were ultimately published, the editors would like to express their appreciation and thank the following reviewers for the time and dedication that they have shown *Healthcare*:
Aalap ChokshiKhaled HosnyAamir JalilKhaled TrabelsiAaron M. WendelboeKhalid SiddiquiAbad KhanKhalim KhujamatovAbbas MardaniKhattak NisarAbbas SmileyKhushwant Singh BhullarAbbas Ullah JanKichang LeeAbbirami ElangovnKiessoun KonatéAbdalkarem AlsharariKihong HongAbdelhaleem KhaderKijin KimAbdelhameed IbrahimKim BuchholtzAbdollahyan MaryamKin Kui LaiAbdul Rashid AzizKinga GawelAbdulaziz AhmedKinga KimicAbdulellah Al ThobaityKip ThomasAbdulkadir AtalanKiran KunduAbdulkarim HasanKiran Maini GerhardssonAbdullah AlhifanyKishore BalasubramanianAbdullah AssiriKi-Sun LeeAbdullah BayramKitamura MineakiAbdulqadir J. NashwanKit-Lun YickAbeer ElshaterKlaus GratzAbel Bauero EscribanoKlaus J. WirthAbel LermaKlaus-Dieter WerneckeAbhijit MondalKoji SawadaAbhijit PakhareKoji YamatsuAbhishek DeyKondapa Naidu BobbaAbhishek KaushikKonosuke NakajiAbhrajit GangulyKonstantinos ChristopoulosAbid HussainKonstantinos KafetsiosAbigale T. MatulewiczKonstantinos LimniotisAbraham Bernárdez GómezKonstantinos TsaknakisAbraham PouliakisKornanong YuenyongchaiwatAchmad Nizar HidayantoKornelia BatkoAdam WichniakKornelia ZarębaAdam WiśniewskiKosho YamanouchiAddi Ait-MloukKossakowska KarolinaAddisson SalazarKosuke OkuAdebayo AdeyinkaKota BokudaAdebayo AgbejuleKota KodamaAdebowale OgunjirinKou KitabayashiAdel A. BahaddadKoulla ParpaAdel RazekKoumantakis GeorgeAdela BadauKourosh SheibaniAdelaide Ferreira Leite MartinsKousalya PrabaharAdelle McArdleKoustav SarkarAdilson MarguesKoyin ChangAdilson MarquesKranti BhavanaAdina NegreaKrasimira Borislavova IvanovaAdina ThomaidisKrassimir MarkovAdina Turcu-StiolicaKrishna Mohan SurapaneniAditi KulkarniKrishnan GanapathyAditya WaghKrisna PiravejAdnan AynaKristen N. SobbaAdnan HaiderKristen R. BreitAdnan Muhammad ShahKristian LeisegangAdnan Shami ShahKristian V. JonesAdrià ArboixKristina MeyerAdrian BuzeaKristine HeidemeyerAdrian CurtoKrisztián GáspárAdrián Varela SanzKrystian TuczyńskiAdrian Vasile MuresanKrzysztof DowgierdAdrian Zaragoza-BastidaKrzysztof J. CzarnockiAdriana Caldo-SilvaKrzysztof JurekAdriana CeciKrzysztof KarszniaAdriana GrigorașKrzysztof KowalAdriana HenriquesKrzysztof KusAdriana M. CalvoKrzysztof Sas-NowosielskiAdriana MikaKrzysztof ZdziarskiAdriano FriganovicKuangming KuoAdrienne K. SalmKuei-Mei ChengAekkhaluck IntharuksaKun WangAfsane RiaziKun YangAfshan BeyKunal AjmeraAfzal ShaikKunal N. PatelÁgata AranhaKundlik GadhaveAgata BalińskaKun-Hua LeeAgata Carmela NicolosiKuo-Chou ChiuAgata M. SocciniKuo-Jung LeeAgata OrzechowskaKuoming LeeAgata StanekKurt MarquardtAgilandeeswari LoganathanKurt SvärdsuddAgnė BrandišauskienėKwang-Sig LeeAgnė UlytėKwong Nui SimAgnes PeterfalviKwua-Yun WangAgnese De MarioKyoungrim KangAgnieszka BieńKyu Young ChoiAgnieszka ChwałczyńskaKyudong ParkAgnieszka JastrzębskaKyu-Nam KimAgnieszka KamińskaKyung Eun LeeAgnieszka Kozioł-KozakowskaKyung Hwan KimAgnieszka KulikKyung Rae KoAgnieszka LasotaKyungchul SongAgnieszka Lecka-AmbroziakKyungyeol (Anthony) KimAgnieszka ŁyśKyung-Yong ChungAgnieszka MlynarskaKyuseok KimAgnieszka NawrockaL. Douglas RiedAgnieszka Owczarczyk-SaczonekLadislav BatalikAgnieszka ŚliwkaLadislav ŠtěpánekAgnieszka StawarskaLaia DomingoAgnieszka TruszkowskaLaima SapezinskieneAgnieszka WerenowskaLaisa Liane Paineiras-DomingosAgnieszka Wojewódzka-WiewiórskaLaith AlzubaidiAgnieszka WoźniewiczLaith T. KhraisAgnieszka ZagórskaLalitha GummidiAgnieszka Zelek-MolikLambert SpaanenburgAgustín Aibar-AlmazánLambrini KourkoutaAgustín Díaz-ÁlvarezLamiaa M. A. AliAgustín Llopis-GonzálezLampropoulou SofiaAgustín Rodríguez-EstebanLana MinshewAh YusufLana NežićAhmad AfifyLandy LuAhmad AlbaghdadiLara BaticicAhmad JamlehLara KularAhmad Samed Al-AdwanLara MottaAhmed AbdeenLarbi BoubchirAhmed AliLars ClemmensenAhmed ElamragyLatefa DardasAhmed GalhoumLaura A. DowneyAhmed ImranLaura Antonio-ZancajoAhmed Jibril AbdiLaura Aracely Contreras-AnguloAhmed KarmaouiLaura BavaAhmed S. SaidLaura C. Sánchez-SánchezAhmet AyazLaura CannavòAhmet Fatih TabakLaura CerviAhmet KoçLaura CocoAhmet TanhanLaura FreemanAhsan JavedLaura FroehlichAhsan UllahLaura J. RushAi Ni TeohLaura LaffranchiAida MetoLaura María CompañAida PetcaLaura Muñoz-BermejoAikaterini MantakaLaura NathansAimee M. DiazLaura SaundersAimen KhacharemLaura Teresa Hernández-SalazarAina Torrejon-MoyaLaura TimmsAinhoa Ulibarri OchoaLauren FowlerAinoa Roldán AliagaLauren Miller-LewisAisha NazirLaurence J. WalshAitor Garay-SánchezLaurence Simard-ÉmondAjay PalLaurent ChatreAjinkya PawarLaurent DonzéAkbar NawabLaurent SuppanAkhil Jabbar MeerjaLauri SoinneAkhila VeerubhotlaLaVerne L. BrownAkhtar AkhtarLavinia MoleriuAkihiko HiyamaLayth Mula HussainAkihiko KatayamaLeandro Dias Da SilvaAkihiro TamuraLeandro José Rodrigues MachadoAkikazu ShinyaLechosław Pawel ChmielikAkinobu UsamiLechuang ChenAkira MonjiLee Ann Laurent-ApplegateAkshay GoelLee BartelAlaa Din AbdinLee NguyenAlan HuebnerLee Yue-PingAlarico ArianiLee-Kuen ChuaAlba Viana LoraLei WanAlbert F. SmithLei ZhangAlbert RossiLei ZhouAlbert SabbanLeijian YongAlbert Thomas AnastasioLeila GhahremaniAlbert YeungLeili TapakAlberto Adanero VelascoLena FrancescoAlberto ChighineLena HofmannAlberto CiprianiLena WimmerAlberto FantinLenin PavonAlberto Frutos Pérez-SurioLeonard Christopher SchmeelAlberto García GómezLeonardo BocchiAlberto González-GarcíaLeonardo Catalano IniestaAlberto ModeneseLeonardo Juan Ramirez LopezAlberto Quílez-RobresLeonardo ManciniAlberto Rodríguez-ArchillaLeonardo PellicciariAlberto SalomoneLeonel PrietoAlberto SardiLeonie HalloAldo RoccaLeonzio FortunatoAldona PiwkoLesley CharlesAlejandra Alonso-CalveteLevon GevorkovAlejandra Giselle Juárez-RebollarLewis HsuAlejandro Canedo-GarcíaLia E. TsotsosAlejandro De La ParteLiana Claudia SalanţăAlejandro Heredia CiuroLiana RichardsonAlejandro Martínez-RodriguezLiang YeAlejandro Roman LoeraLiang-Ching ChenAlejandro TelloLiang-Jen WangAleksander DokollariLianyong QiAleksander KobylarekLiborija Lugović-MihićAleksandra Gaworska-KrzemińskaLi-Ching ChangAleksandra JarońLida FanAleksandra Karczmarska-WódzkaLidia GavicAleksandra KovačevićLidia PerencAleksandra KrólikowskaLigia RusuAleksandra Milic LemicLikwang ChenAleksandra RadeckaLilach SoreqAleksandra RogowskaLilanthi BalasuriyaAleksandra RyłLilia LesykAleksandra S. KristoLilian Genaro Motti AderAleksandra StupakLilian SosaAleksandra SzylińskaLiliana DumitracheAleksandra Wisłowska-StanekLiliana FeleagaAleksandra ŻebrowskaLiliana MazzaAlena FurdovaLiliana MontellaAlessandra De MatteisLiliana PitachoAlessandra GiordanoLiliana Veronica DiaconescuAlessandra PatronoLiliya ZiganshinaAlessandra Verzelloni SefLilla MakszinAlessandro AgostiniLilli WinterAlessandro AnselmoLin ZhuAlessandro ComandoneLina Díaz-CastroAlessandro De SireLinbo ZhaoAlessandro FailoLincai YeAlessandro FeolaLinda BashirAlessandro FratiLinda BirtAlessandro MantiniLinda J. Larson-PriorAlessandro MassaroLinda JonesAlessandro Mauro TavoneLinda K. RauschAlessandro NotaLinda Katherine JonesAlessandro RodolicoLinda KeilmanAlessandro ScalaLinda PetersAlessandro TelLinda SaraivaAlessandro TestaLinda WatsonAlessandro VittoriLindawati S. KusdhanyAlessandro VizzarriLindsay M. McCaryAlessia D’AndreaLindy M. RossowAlessia GrigolettoLing TangAlessia RenziLing YinAlessio ArdizzoneLinh N. HoangAlessio CampisiLinlin HuAlessio ContiLinping ZhaoAlessio Danilo ComisLinus Mabughi NyiwulAlessio RocconLionel Sanchez-BolivarAlessio TurcoLiping XieAlex Buoite StellaLirong WangAlex HanLisa BauleoAlex Harley CrispLisa HaleAlex NiuLisa LandgrafAlexander A. PugovkinLisa LungaroAlexander BerezinLisa M. GallagherAlexander GardnerLiudmila Gerasimova-MeigalAlexander GorokhovskyLiudmila LiutskoAlexander KorthausLiv Wergeland SørbyeAlexander NortaLiverana LaurettiAlexander OberhuberLiviu MoldovanAlexander S. NovikovLixing WangAlexander SchultzeLiz SimonAlexander SmirnovLiza LeeAlexander VodyahoLiza M. ConyersAlexandra AgostiniLjudmila StojanovichAlexandra González AguñaLluis OviedoAlexandra Ioana MihăilescuLon Van WinkleAlexandra LappasLong Chiau MingAlexandra LopesLopetcharat KannaponAlexandra MartuLoredana IvanAlexandra PereiraLoreen ThürmannAlexandra RomanLorena Caridad Y. López Del RíoAlexandra Vink-NieseLorena CharrierAlexandre González-RodríguezLorena DucaAlexandre MalekLorena Garcia Del RioAlexandre Morais NunesLorena Gutiérrez-HermosoAlexandre Wesley Carvalho BarbosaLorena Tarriño-ConcejeroAlexandros ArgyriadisLorenza VantaggiatoAlexandros G. SykarasLorenzo AngeliniAlexandros LaiosLorenzo BrognaraAlexandru BucurLorenzo CostaAlexandru Florin RogobeteLorenzo FaggioniAlexandru Gabriel BejinariuLorenzo FerriAlexandru Silvius PescariuLorenzo FranceschettiAlexandru UliciLorenzo GianquintieriAlexey SarapultsevLorenzo LippiAlexis TalbotLorenzo RusticoAlfonsina D'IorioLorenzo VigentiniAlfonso Alvarado-LorenzoLoreta Buksnyte–MarmieneAlfonso García-MongeLorie RichardsAlfonso Gutierrez-SantiagoLoris NanniAlfonso Isidro López DíazLothar FunkAlfonso Martínez-MorenoLouie Mar A. GangcuangcoAlfonso Martínez-NovaLouis ArnouldAlfredo BrunoLouise BuckleyAlfredo Cruz-GregorioLouise MobergAlfredo G. CasanovaLoukas ChatzisAli AbbaraLoukianos S RallidisAli AhmedLourdes María Fernández SeguínAli Al KaissiLovorka BrajkovićAli BoolaniLoza Carlos MastachiAli CaykoyluLu LiAli JahaniLuca BuzzattiAli Kerim YılmazLuca CegolonAli Mohammad AlqudahLuca GallelliAli MontazeriLuca Giovanni LocatelloAli NadzalanLuca NicosiaAli RostamiLuca OngaroAli Sadegh-ZadehLuca Oscar Redaelli De ZinisAlia TelliLuca PetrignaAlica HokynkováLuca RussoAlice BaranaLuca SimioneAlice Elena GheneaLuca TestarelliAlice GiontellaLucas De Oliveira TeixeiraAlice Kit Ying ChanLuci MartinAlicia FournierLucia A. SealeAlicia Martínez-RodríguezLucia BottiAlicja KalusLucia GalvagniAlicja KamińskaLucia LombardiAlina CernasevLucia MangoneAlina Delia PopaLucia MuracaAlina Petronela HallerLucia Princiotta CariddiAlireza AbbasimoshaeiLucia StančiakováAlireza Nikbakht NasrabadiLucia TattoliAlisa WrayLuciana LabancaAlison WilliamsLuciane Viater TureckAlix YoungLuciano Rodríguez-DíazAlka BishnoiLúcio Fernandes FerreiraAlla KravetsLúcio FrigoAlla LubovichLucio MangoAllam Jaya PrakashŁucja Zielińska-TomczakAllysson Allex AraújoLucrezia BottalicoAlok SutradharLucrezia Ginevra LulliAlon FarfelLucy BregmanAlongkorn PimpinLucyna ŚcisłoAlonso Alonso-AlonsoLudmilla ChinenAlper SozutekLudwig ApersAlpo VuorioLuigi BonavinaAltaira D. DearbornLuigi BruscianoAltti NäsiLuigi La ViaAlvarado-Ibarra MarthaLuigi LoscoÁlvaro Clemente VivancosLuigi MaranoAlvaro García Del CastilloLuigi MeccarielloAlvaro Hermida-AmeijeirasLuigi RuggieroAlvaro Lopez-EscamillaLuigi TritapepeAlvisa PaleseLuis Albendín-GarcíaAlyssa HuffLuis Alberto Gaitán-CepedaAlzira MotaLuis Andreu CaravacaAmador Jesús Lara SánchezLuis AnibarroAmaia YurrebasoLuis Ceballos-LaitaAmal A. Al-ShargabiLuís FaíscaAmal PereraLuis FernandezAmalia CorneaLuis Fernando MachadoAmalia Sillero-SilleroLuis JunqueraAmand FührerLuis Miguel Dos SantosAmanda IglesiasLuís MonteiroAmanda J. ChaseLuis N. CoriaAmanda WilsonLuis Rodrigo MartínAmani Yousef OwdaLuis Salvador-RodríguezAmany MohamedLuis SilveiraAmar KanekarLuís TinocaAmbar Lopez-MacayLuis Valero AguayoAmbrina A. QureshiLuis Villalobos-GallegosAmeesh M. IsathLuísa Bandeira LopesAmelia DíazLuisa OrrùAmi RokachLuiz NakamuraAmina NasriLuiz Palucci-VieiraAmine ElbouzidiLuiz Peres-NetoAmir HadiLuiza PiersialaAmira Mohammed AliLuk JimAmirul MukmininLuka MatakAmit ChandelLukas FischerAmit GoelŁukasz A. PoniatowskiAmit K. SinghŁukasz CichockiAmit PantLukasz JaskiewiczAmit PorwalLukasz LapajAmitava MukherjeeŁukasz MałekAmjad Islam AqibŁukasz NawackiAmjad KhanŁukasz PałkaAmmar AltemimiŁukasz RydzikAmr FoudaŁukasz RypiczAmr HassanLukasz SzarpakAmrendra MishraŁukasz WirkusAmrit MukherjeeŁukasz ZapałaAmrita PaiLuke BalcombeAmulya YaparlaLuke Del VecchioAmutha RamadasLuminiţa MoraruAmvrosios OrfanidisLuminita OanceaAmy A. Clements-CortésLunda ShenAmy FehlLut TamamAmy MooreLu-Ting KuoAna AdanLutz NährlichAna AndradaLuyen VuAna BankoLuyu XieAna Beltrán VelascoLuz Adriana Aristizábal BecerraAna CastanhoLuz Zas VarelaAna CoelhoLyda G. GarciaAna Corte-RealLykourgos MagafasAna DascaluLynette HodgesAna Diez-IzquierdoLynn LepplaAna Filipa PoeiraLynn M. PezzaniteAna Gentil-GutiérrezLynne HampsonAna I. Álvarez-MercadoM. IlayarajaAna IvaniševićMa De Los Ángeles Merino GodoyAna KezićMaayan ShachamAna Lúcia TerraMabel VazquezAna Luísa RodriguesMaciej ChęcińskiAna Manzano-LeónMacIej PatrzykAna Maria Da Palma MoreiraMaciej SalagierskiAna María Salinas-MartínezMaciej SikoraAna María Vázquez-CasaresMaciej ŻukowskiAna Paula AyresMadalina-Gabriela IliescuAna Rita SilvaMaddalena CannitoAna RuigomezMadeeha MalikAna Sofia Ruivo AlvesMadelon L. FinkelAna Sofia VinhasMadepalli LakshmanaAna Teresa Corte-RealMadhan JeyaramanAna TomasMadhavan NampoothiriAnabela Salgueiro-OliveiraMaebh HardingAna-Isabel Corregidor-SánchezMagda RiveroAnamaria BechirMagdalena Graczyk-KucharskaAna-Maria CazanMagdalena HurkaczAnamaria CiubaraMagdalena IorgaAnamarija Jurčev SavičevićMagdalena KołakAnand NayyarMagdalena KorżyńskaAnanda SidartaMagdalena KowalówkaAnandakumar HaldoraiMagdalena LisAnanth Kumar KammalaMagdalena Mackiewicz-MilewskaAnantha HarijithMagdalena Mazur-MileckaAnanto AlhasyimiMagdalena MititeluAnastasia G. MitseaMagdalena SzopaAnastasia PapadimitriouMagdalena WyszynskaAnastasia ProdromidouMagdalena ZadwornaAnca Adriana ArbuneMagnus SvartengrenAnca Chelariu-RaicuMahdi ZavvarAncy JosephMahesh BavineniAndi RrokuMaheswari MuruganandamAndoni Carrasco-UribarrenMahmoud A. MossaAndras BanvolgyiMahmoud AbulmeatyAndras BikovMahmoud ElbattahAndrás FittlerMahmud HasanAndré CarneiroMahyar Habibi RadAndré L. L. BachiMaija Huttunen-LenzAndre NienaberMainul HaqueAndré NohlMaitreyee WairagkarAndré OliveiraMaja BaretićAndré Pimenta FreireMaja MiskulinAndré RamalhoMaja SurbatovicAndre RodackiMajdy IdreesAndré SilvaMajed AlamriAndre WirriesMajid Mufaqam Syed-AbdulAndrea BotturiMajid Rasool KamliAndrea ButeraMakella S. CoudrayAndrea CaraiMakkai-Popa Silviu-TiberiuAndrea ChiricoMakoto TsukaiAndrea CiminiMakram SouiAndrea Clemente PalmierMalak El-HazmiAndrea De VitoMalcolm KooAndrea DincherMalene PedersenAndrea FabboMalgorzata DobrzynskaAndrea FaggianoMałgorzata Lesińska-SawickaAndrea GiansantiMałgorzata Mikaszewska-SokolewiczAndrea Giuseppe MaugeriMałgorzata MoszakAndrea JaníkováMałgorzata PasekAndrea KenkmannMałgorzata Peregud-PogorzelskaAndrea L. HildebrandMalgorzata PihutAndrea Martín-VacasMalgorzata WojcikAndrea Milostić SrbMałgorzata Zegan-BarańskaAndrea MininiMalshani L. PathirathnaAndrea Paola Rojas GilMalvinder S. ParmarAndrea PintorMa'mon M. HatmalAndrea SambriMamoon AldeyabAndrea SzékelyMamoon RashidAndrea TittarelliMamoru KusakaAndrea TrevisanMamoun AbuhelouAndréanne SharpManabu MaedaAndreas ÄlgaManal MohammedAndreas IhleMandy SteimanAndreas LipphausManela GlarcherAndreas PolydorouManfred BornewasserAndreas WarnkeManfred SchmittAndree HartantoManh Tung HoAndreea AvasiloaieiManikandan RamachandranAndreea Claudia SerbanManikant TripathiAndrei C. HolmanManish Vijaya KumarAndrei HolmanManisha NigamAndrei ShpakouManlio SantilliAndreia GarcêsManoj Kumar TembhreAndreia LimaManoj RamanathanAndrej ThurzoManoj SharmaAndrés Alonso Agudelo-SuárezManolis ManioudisAndrés Barrios-RubioManon LemondeAndrés Fernando Avilés-DávilaMansi SharmaAndrés García-GómezMansokku LeeAndrés Gené SampedroMansour A. MahmoudAndrés Pastor FernándezManuel Casal-GuisandeAndres Reinoso-CoboManuel Coheña-JimenezAndrew B. RosenblumManuel De La SenAndrew Chih Wei HuangManuel Durán-PovedaAndrew FreedmanManuel Filipe Pereira Cunha Martins CostaAndrew FribleyManuel Florindo Alves MeirinhosAndrew J. LarnerManuel FrancoAndrew M. DylagManuel García-SilleroAndrew M. PickeringManuel Jesús-AzabalAndrew ObergManuel Pabón-CarrascoAndrew PoveyManuel PereiraAndrew RuysManuel Rebollo-SalasAndrew StrongManuel Sánchez-MollaAndrey D. NasledovManuel SorianoAndrija MatetićManuel StruckAndrius KlimašauskasManuel Vargas-VargasAndrzej HeckerManuela Adelinde AschauerAndrzej JanowskiManuela Expósito-RuizAndrzej JarynowskiManuela MeirelesAndrzej KicińskiManuela SilvaAndrzej PiotrowskiManvi SharmaAndrzej SemczukMaolin LuAndrzej SkrętMara ClementeAndrzej TomczakMara EyllonAndrzej TukiendorfMarc DautyAndrzej ZankiewiczMarc JacquinetAndy PetroianuMarc WeinsteinAndy Wei-Ge ChenMarc ZanelloAne Murueta-GoyenaMarcel CopperAneta GrochowskaMarcel HanischAneta Ptak-ChmielewskaMarcela KanovaAneta R. BorkowskaMarcela Petrová KafkováAneta RadziwonMarcela Sefora NemteanuAneta WoźniakMarceli ŁukaszewskiAngel De JuanasMarcelino Pérez-BermejoAngel López-GonzálezMarcell CsanádiAngel Oliva Pacual-VacaMarcello CherchiÁngel Vicario-MerinoMarcello Della CorteAngela AmmirabileMarcello Di MartinoAngela Beale-TawfeeqMarcello SariniÂngela GonçalvesMarcelo CoertjensAngela GuarinaMárcia BarrosAngela MertigMarcin DerwichAngela RepanoviciMarcin GierachAngélica Angélica Bulcão Portela-LindosoMarcin GierekAngélica Juárez-LoyaMarcin JozwikAngeliki AngelidiMarcin MiłkowskiAngelo B. HookerMarcin MorońAngelo CapodiciMarcin RudzkiAngelo CardarelliMarcin WnukAngelo Di IorioMarcio Costa De SouzaAngelo MontanaMarcio ValkAniello MaieseMarco Antonio Hernández-LepeAnis KoubaaMarco BatistaAnish MukherjeeMarco DettoriAnish ShivaramMarco Di SarnoAnita A. DaviesMarco Echeverria-VillalobosAnita Jagroop-DearingMarco EspositoAnita KovácsMarco FarronatoAnita MajchrowskaMarco FerriseAnita PipereMarco FioreAnita SłupskaMarco La VerdeAnita SubramanianMarco MeyerAnjul KhadriaMarco NisiAnkit GuptaMarco PalumboAnkita PunethaMarco PortelliAnn HardyMarco SapienzaAnn LargeyMarco TjioeAnn SvenssonMarco Trabucco AurilioAnna AugustynowiczMarco TuonoAnna B. FuksMarcos BrioschiAnna Babicka-WirkusMarcos Marcos MichaelidesAnna BagieńskaMarcos Mecías-CalvoAnna BartosiewiczMarcus D. LanceAnna BednarekMarcus StoetzerAnna BeniniMardiati NadjibAnna BirkováMaree SimpsonAnna BocchinoMarek KarkulaAnna Bogusława Pilewska-KozakMarek KiliszekAnna BroschMarek KruszewskiAnna BrzeckaMarek MiloszAnna BukowskaMarek MotykaAnna DanielewiczMarek NahajowskiAnna De FalcoMarek SawickiAnna Drelich-ZbrojaMarek SzelągowskiAnna FlorowskaMaresca Attard PizzutoAnna Gárate EscamillaMargalida Coll-AndreuAnna GavrilovaMargaret F. KeilAnna HartonMargaret SherburnAnna Janas-NazeMargareta TeodorescuAnna JędrzychowskaMargarete SteinerAnna Jodejko-PietruczukMargarida SaraivaAnna Kasielska-TrojanMargarita A. MorozovaAnna KawalecMargarita Gozalo DelgadoAnna KittaMargarita StankovaAnna Kołodziej-ZaleskaMargherita PallocciAnna LeśkówMargot W. M. De WaalAnna MajdaMaría A. Pérez-HerreroAnna ManolopoulosMaria Alexandra PanăAnna Maria BianchiMaria Alexandra PapadimitriouAnna Maria GianniniMaria Andrée López GómezAnna Mazurek-KusiakMaria Angeles Caballero-MoraAnna MertasMaria Angeles Martinez RuizAnna Michalak-StomaMaría Antonia Parra-RizoAnna MichalikMaria António CastroAnna MnatsakanovaMaria ArmaouAnna N. BerlinaMaria BarsanAnna OlczakMaria Blanco-DiazAnna Paradowska-StolarzMaría Camacho EncinaAnna PiekarskaMaría Carrillo-DíazAnna PodlasekMaria CassarAnna ProkopiakMaria Chiara CascheraAnna RogalskaMaria Chiara GattoAnna RosofskyMaría Cristina Martínez-FernándezAnna RozensztrauchMaria DaglaAnna Rybarczyk-SzwajkowskaMaria De Fátima Pereira Da SilvaAnna SiemiątkowskaMaría Del Carmen García-MendozaAnna SobiepanekMaría Del Carmen Pérez-FuentesAnna StankowskaMaría Del Carmen Valls MartínezAnna StarshinovaMaría Del Henar Pérez-HerreroAnna StarzyńskaMaría Del Mar Fernández-MartínezAnna Stefanowicz-BielskaMaría Del Pilar Salas-ZárateAnna TychmanowiczMaria Do Céu Patrão NevesAnna Tyrańska-FobkeMaría Dolores Sánchez FernándezAnna Vila-MartíMaria Dos Anjos DixeAnna ZellmaMaria Dyah KurniasariAnnachiara CavaliereMaria Elena NenniAnna-liisa TammMaria Elena Vila-SuárezAnnalisa Di BernardinoMaría Emilia SantillánAnnalisa GrandiMaria FaulknerAnnamária PallagMaría Ferández-MartínezAnne Christine WoolridgeMaría Fernanda Bernal-OrozcoAnne KuhntMaria FortofoiuAnne KuusistoMaria Francesca PiazzaAnnemarie Van Der WalMaria Francesca RossiAnnie George-PuskarMaria Gabriella VersoAnnika Helgadóttir DavidsenMaria GacekAnoop KumarMaria GiannìAnoop SheshadriMaria Giulia MariniAnroop NairMaria Giuseppina BartoloAnselm JordaMaría González Cano CaballeroAnthony FardetMaria GrajdianAnthony L. KovacMaría Granados-SantiagoAntje Büttner-TeleagăMaria Grazia Lourdes MonacoAntoine NaemMaría Guadalupe Frías-De-LeónAnton KiselevMaría Isabel Mármol-LópezAnton S. TkachenkoMaría Isabel Parra ArévaloAnton S. TsybkoMaría Isabel Tovar-GálvezAntonella SmeriglioMaría Jesús HerreroAntònia Costa-BauzáMaria Jesus Vinolo GilAntonia M. CaleyaMaría José Bautista-LlamasAntonia PelegrínMaría José de Dios-DuarteAntonija TadinMaría José Flores TenaAntonije OnjiaMaria José Lumini LandeiroAntonina ArgoMaría José Pérez LacastaAntonino FotiaMaria José SousaAntonino ManiaciMaria KapritsouAntonino MazzoneMaria LavdanitiAntonino MorabitoMaria Luisa IndianaAntonio AmmendoliaMaría M. Morales Suárez-VarelaAntonio AquinoMaria Maddalena MarrapodiAntonio BellastellaMaria MalliarouAntónio CaleiroMaria MazzitelliAntonio Cartón-LlorenteMaría MeloAntonio CicchellaMaría Mendoza-MuñozAntonio CoboMaria Mihaela GrajdianAntonio Daniel García RojasMaría MoralesAntonio DuranMaria MorelloAntónio Fernando Salgueiro AmaralMaría Navarro-GranadosAntonio Francesco Maria GiulianoMaria NóbregaAntonio GiordanoMaría Orosia Lucha-LópezAntonio Granero-GallegosMaria Otília ZangãoAntonio Jesús Molina FernándezMaria PaparoupaAntonio LeoMaria Petronilla PennaAntonio LloretMaria RubegaAntonio Luque-De la RosaMaria Salvina SignorelliAntonio M. Burgos-MolinaMaria SarapultsevaAntónio Manuel Neves VicenteMaria SofologiAntonio Martinez-SabaterMaria T. Van HartenAntónio Miguel MonteiroMaria Tarico Bico IsabelAntonio MolinoMaria Teresa GiglioAntonio Ranchal SánchezMaria Teresa InzitariAntonio RomanelliMaria Teresa Murillo-LlorenteAntonio Sarasa CabezueloMaría Teresa Solís-SotoAntonio Sarria-SantameraMaría Vanessa VillasanaAntónio SousaMaría Vázquez-GuimaraensAntonio TufanoMaría Yolanda Castellote-CaballeroAntonio VallanoMaría-Arántzazu Ruescas-NicolauAntonio Vicente Rodríguez FuentesMaría-Carmen Sánchez GonzálezAntonio-Jesús Marín-PazMariachiara IppolitoAntonios GasteratosMaria-Do-Céu MonteiroAntony G. PhilippeMaría-Leticia Meseguer-SantamaríaAnuchit JitpattanakulMarian AláezAnuli NjokuMarian WoźniakAnupam MukherjeeMariana CernicovaAnurag SatpathyMariangela AlbertiniAnusha PenumartiMariann HarangiAnusna ChakrabortyMarianna BellafioreApostolos PapachristosMarianna LeopoulouAppel MahmudMarianna MuravyevaAranzazu DuqueMariano CingolaniAras BozkurtMaría-Pilar Molina-TorresArcady PutilovMariarosa GiardinaArchana SaxenaMarie BarnardAreti StavropoulouMarie De PerioArfan GhaniMarie SjögrenArghya DattaMarie-Luise KromreyArgyro PachiMarien GrahamAriadna Hernaiz-SanchezMarija GredicAriane SchwankMarija IvanovArif MandanganMarija JevticArijit DasMarija MeznaricAristidis GaliatsatosMarika ComegnaAriuntuul GaridkhuuMarilia PauloArjun SapkotaMarina KarpenkoArkadiusz JasińskiMarina PiscopoArkadiusz ZurawskiMarinella CocoArkady KotlyarMário AntunesArkalgud RamaprasadMario CaggianoArlette Suzy SetiawanMario FranchiniArmanda PereiraMario JimenezArmando AliuMario José Costa De MacedoArmando CaseiroMario MontaninoArmando Silva-AlmodóvarMario TabaArminas JasionisMário W. L. MoreiraArnaud PagesMariola BorowskaArnulfo Ramos-JiménezMariola DrozdArpita RaiMariola GłowackaArrate LasaMarion MuchaArsenio PaezMarion PorcherieArthur De Sá FerreiraMarion RussellArthur FerreiraMariona RiusArthur MolnarMarios ConstantinouArtur GłuchowskiMarios SpanakisArtur Jorge FerreiraMarius RademakerArtur PasternakMariusz DuplagaArtur StruzikMariusz LuterekArun Kumar RayMariusz MalinowskiArun Pandian J.Mariusz WysokińskiArunachalam ThirunavukkarasuMarja Silén-LipponenArzu BeklenMárk AntalAsad KhanMark BrookeAsad UllahMark Elisabeth Theodorus WillemsAsaf AchironMark K. TranstrumAsaf BilgoryMark PalmerAsanka BulathwattaMark ReybrouckAscensión Doñate-MartínezMark S. FleisherAscensión Palomares RuizMark SchiffmanAsghar JahanshahiMark V. PaulyAsha Jyothi SiddappaMark W. LuskAshish JainMarko BaškovićAshish NoronhaMarko PericAshkan RashadMarko VuletićAshokkumar ThirunavukkarasuMarkus GessleinAshril YusofMarkus PuchingerAshuin Kammar-GarcíaMarleide Da Mota GomesAsif KhaliqMarlena RobakowskaAsmaa Mohammed HassanMaroof AlamAsrat AmnieMaroš HrubinaAssunção NogueiraMarquisette Glass LewisAsta SavanevičienėMarta Amor-BarbosaAsunción M. MayoralMarta BiegańskaAteeq Ur RehmanMarta BistronAtefeh VaeziMarta CavigliaAthanasia PapazafiropoulouMarta DziechciarzAthanasia PatakaMarta Elena Losa IglesiasAthanasia PrintzaMarta GimunováAthanasios KiourtisMarta Jeruszka-BielakAthanasios N. TsartsalisMarta KopańskaAthanasios SachlasMarta Makara-StudzińskaAthikhun SuwannakhanMarta MaleszaAthina A. SamaraMarta María Hernández-MartínAthina TsanousaMarta MatosAtiqul Haq MazumderMarta MuszalikAtiyeh AbdallahMarta Pereira AlvesAtoany Nazareth Fierro RadillaMarta RachelAtsushi SuzukiMarta Regina Cezar-VazAttila FrankoMarta Rodríguez-HernándezAttila KissMarta RusnakAttila TothMarta WłodarczykAtukuri DorababuMarta ZubiaurAudrey GuenicheMartha Alejandra Morales-SánchezAudrey J. MurrellMartha-Spyridoula KatsarouAudrey Opoku-AcheampongMartin CorstenAugusto FuscoMartin Oliver SchmiadyAugusto PalombiniMartin PecAuliya A. SuwantikaMartín Pérez-SantosAurel BurciuMartin RakusaAurel PeraMartin Sanchez-GomezAurel Popa-WagnerMartin SchepelmannAurelia OstrowskaMartin VlkAurelie GoncalvesMartin ZiegerAurelija BlaževičienėMartina F. BaumannAurora GonzálezMartina Fernández GutiérrezAvi MagidMartínez CastelaoAviad Tur-SinaiMartis GeorgianaAvinash R. PatwardhanMartti VastamäkiAvishek ChoudhuryMartyna Kasper-JędrzejewskaAxel SteigerMaruti Ram GudavalliAya FujiwaraMarvin A. Soriano-UrsúaAyataka FujimotoMarwa ElgendyAyaz ShahidMarwan A. S. BukhariAysegul NalcaMarwan OsmanAyush DograMary BarretoAzam AslaniMary FindlingAzhar ImranMary HiddeAziz A. FallahMary KamienskiAzumi IshizakiMary Katherine HoyB. N. Pavan KumarMary Kathleen ClarkBabu R. DawadiMary L. WhiteBach Q. HoMary LashleyBadar UmarMaryam FarooquiBadau AdelaMarzulli MicheleBahai JiaoMasa SasagawaBalachandar VellingiriMasahiko TsuchiyaBalazs HankoMasahiro TakahashiBarbara BaranowskaMasahiro YoshikawaBarbara D’AmenMasahito FushimiBarbara GordonMasakiyo YatomiBarbara MognettiMasanao NakamuraBarbara PieperMasanori HashimotoBarbara ProbierzMasaomi IkedaBarbara RemberkMasatsune TsujiokaBart PijlsMasayoshi TanishitaBart RuttemanMasoud MadadelahiBart VisserMasoumeh Kourosh AramiBartłomiej GmajMassimiliano ManciniBartłomiej SoltysikMassimiliano Matteo PellegriniBartłomiej SzulczykMassimo BelliniBartolomé Pizà-MirMassimo RalliBaseer U. AhmadMassimo SalvatoriBasil MathewMassimo SalviBassam AyoubMasud RabbaniBassma ElwakilMateja LorberBayram CeylanMateus Coelho SilvaBayu Adhi TamaMateusz BabickiBayu Satria WiratamaMateusz GliwińskiBeat GöpfertMateusz GrajekBeata BucheltMateusz JankowskiBeata HoffmannMateusz KoteckiBeata Mrozikiewicz-RakowskaMateusz KowalBeata PiȩtaMateusz KuklaBeata SperkowskaMateusz MarciniakBeata SzluzMateusz RozmiarekBeatrice AlbanesiMateusz ZawadkaBeatrice Gabriela IoanMatevž PesekBeatrice LeonardiMathieu DelormeBeatrice ThielmannMathilde SengoelgeBeatrix Krause-SorioMatias NollBeatriz Álvarez-EmbarbaMats ErikssonBeatriz PérezMatteo Alessio ChiappediBeatriz Rodríguez SánchezMatteo Angelo FabrisBeatriz Servilha BrocchiMatteo BolcatoBebiana CondeMatteo CarpiBee Kang TanMatteo Cotta RamusinoBeenish ChaudhryMatteo CrippaBegoña EspejoMatteo De PastenaBegona Fonseca VázquezMatteo Della PortaBelem BarbosaMatteo FermiBelen Alonso OrtizMatteo FusagliaBelinda HornbyMatteo GulinoBelinda Pinto-SimõesMatteo MarcacciBelur R. LokeshMatteo MatteucciBen BulmashMatteo Monzio CompagnoniBen ClaytonMatteo PaciniBen ParkinsonMatthew FarrowBenedetta GuidiMatthew J. LinksBeng Kwang NgMatthew J. RegulskiBeni Gomez-ZunigaMatthew Kuan JohnsonBenjamin AnsaMatthew PainterBenjamin Barsties Von LatoszekMatthew S. HerbertBenjamin L. MaughanMatthew StrubBenjamin RollesMatthew ThomasBerna RahiMatthew WaltersBernardo VegaMatthias BelauBernhard RauchMatthias KapischkeBernhard RossMatthias LukasczikBertrand DavidMatthias WilmBeth McManisMattia CappellettiBetty BurstonMatylda SierakowskaBetty ExintarisMaulin PatelBetty Yuen-Kwan LawMaura A. E. PilottiBeverly E. CaninMaureen HansonBeverly HoganMauricio Comas-GarciaBhanu Prakash KollaMauricio Garnier-VillarrealBharti SingalMauricio TanoBhavuk GargMaurizio DelvecchioBhawna DevMaurizio EliaBhola PradhanMaurizio MarchesiniBianca E. ŐszMaurizio RicciBianca Ioana TodorMaurizio RomagnuoloBianca N. JacksonMaurizio SchiavonBidisha RoyMaurizio TaurinoBilal Abu-SalihMauro ArcangeliBimala PantheeMauro Giovanni CartaBingxi YanMauro GiuffrèBinh P. NguyenMauro Henrique Nogueira Guimarães De AbreuBinod AcharyaMauro LombardoBirgit BabitschMauro RagoneseBirgitta KerstisMauro RomanelliBirol TopçuMax Carlos Ramírez SotoBjörn FalkenburgerMax O. StephensonBjörn LantzMaxime PichonBlanca R. LopezMaximilian SienerBlanca Rosa García RiveraMaxwell Peprah OpokuBłażej CieślikMay BisharatBo WangMay Portuguez CastroBo ZhaiMayra Urrea-SolanoBogdan DorofteiMayumi NishiBogusława UrbaniakMayur VirarkarBohdan RoznowskiMayuren CandasamyBojan ĐerčanMazhar Javed AwanBojan MasanovicMaziar YazdaniBojjibabu ChidipiMd. Abdul AwalBoluwaji Ade AkinnuwesiMd. AmiruzzamanBonhyuk GooMd. Dilshad ManzarBonnie L. BalzerMd. Hakimul HaqueBoon Giin LeeMd. MushtaqueBoone Claire ElizabethMd. Nazim UzzamanBoon-Huan TanMd. Nazmul Hasan ZilaniBorek SehnalMd. Shafiullah ParvejBoris B. NiraulaMd. Shiblur RahamanBoris C. Rodríguez-MartínMd. Sofiqul IslamBoris Filipović-GrčićMd. Taohidul IslamBorja Herrero De La ParteMeda-Lavinia NegrutiuBoro ŠtrumbeljMeenal ChaudhariBorrelli PaolaMeer M. ChisthiBo-Wei ZhuMeg E. MorrisBoxuan ZhongMegan E. ParkerBo-Young HongMegan MilotaBożena GullaMegha SharmaBrach PostonMegha ShettigarBran Barral BucetaMeghana DesaiBrandon Lucke-WoldMehdi AlikhaniBrandon NathanMehdi HassanBrandon NokesMehmet GültasBrenda Cabrera-MendozaMehmet SarierBrett BlighMehmet YildizBrian BellMehrdad DadgostarBrian D. LoMehrez ElnaggarBrian GodmanMeifang LiBrian K. FoutchMeiko Jovita Makita TafoyaBrian SengstockMei-Tsz SuBrian SwannMeiyi MaBrianna LarsenMelanie BarbatoBrianna Le BusqueMelanie PowisBrice BalmerMeline KevorkianBridgitte SwalesMelissa Agsalda-GarciaBruce I. RapleyMellisa A. HallBruce WallaceMemik Taylan DasBruce WinstonMeng-Che TsaiBruna EibelMengxi ShenBruna VisniauskasMengyu LiBruno GonçalvesMenno T. C. PruijmBruno José Nievas SorianoMercedes Díaz-RodriguezBruno M. FerreiraMervyn J. EadieBruno Macedo De SousaMi Ryeong SongBryan D. HujsakMiaomiao ShiBryan FullerMicah ZuhlBryan J DonaldMichael A. TaliasBryane MichaelMichael AshBryanne N. BellovaryMichael CampbellBukola G. OlutolaMichael CopeBülent KilitMichael Hames-GarciaBülent TopuzMichael HechtBumhee YangMichael InskipBurak BuldurMichael John NortonBurak YulugMichael Joseph DiñoBuse Ozcan KahramanMichael L. RosinoByoung-Hee LeeMichael LiebmanByung-Hoon KimMichael McCroryByung-Kwan SeoMichael NorbergCalin CorciovaMichael OrmsbeeCamelia DelceaMichael R. LissackCameron G. EstrichMichael RönnlundCamilla PasternackMichael RutzlerCândida SilvaMichaela GoodwinCarina González GonzálezMichail DiakosavvasCarl BeckerMichail KotsapasCarla Matos SilvaMichał BulcCarla SerrãoMichał CaputaCarla Sílvia FernandesMichał CzaplaCarlo D'AscenziMichał GinsztCarlo DindorfMichal JurášekCarlo Giacomo LeoMichal KafriCarlo MingantiMichał KrakowiakCarlos A. M. CarvalhoMichał KulusCarlos Bernal-UtreraMichał KuszewskiCarlos DosilMichal OrdakCarlos DurántezMichał RabijewskiCarlos Fernando MourãoMichał SarulCarlos FerrasMichał T. TomczakCarlos Gonzalez BravoMichal WielechowskiCarlos Hernando GómezMichał-Goran StanišićCarlos Jiménez-CorteganaMichela FerrucciCárlos L. Ayán PérezMichelangelo GuaitoliniCarlos LaranjeiraMichele AltomareCarlos López-de-CelisMichele BertoniCarlos Luque-MorenoMichele CassettaCarlos Manuel Torres AlmeidaMichele CovelliCarlos Martín ArdilaMichele Della VenturaCarlos Méndez-MartínezMichele Di CosolaCarlos Roberto Hall BarbosaMichele GolinoCarlos Roberto ValencioMichele KlainCarlos Ruiz-NuñezMichele MerliniCarlos SalaveraMichel-Edwar MickaelCarlos Salavera BordásMichelle BlackCarlos SequeiraMichelle MellenthinCarly DaleyMichelle T. Bover ManderskiCarme CarrionMichinari HiedaCarmela ConteMieszko WieckiewiczCarmelo Ortega RodríguezMigle BacevicieneCarmelo PirriMiguel A. Montero-AlonsoCarmen CasanovasMiguel A. PrietoCarmen Cipriano-CrespoMiguel A. SimónCarmen ConcertoMiguel Ángel García IzquierdoCarmen Daniela Quero CaleroMiguel Ángel Rojo TiradoCarmen De KockMiguel Angel Serrano RosaCarmen Del Pilar Gallardo MontesMiguel Castelo-BrancoCarmen FigueroaMiguel CastroCarmen GianfraniMiguel Ortiz‐BarriosCarmen Massé GarcíaMiguel SellittoCarmen Moret-TatayMiguel Soares‐OliveiraCarmen NeamtuMiha SkvarčCarmen RamírezMihaela HedeșiuCarmen SavinMihaela ZegreanCarmen Suárez-SerranoMihai CraiuCarmen Zabala BañosMi-Hwa SongCarmen-Gabriela SirbuMi-jung KwonCarmina Castellano-TejedorMika Kondo KuniedaCarminda MoraisMike G. TsionasCarmine NovielloMikhail PyatnitskiyCarmine PiconeMikio HayashiCarol LinMikk JürissonCarol NashMikko NurminenCarol Yen Chin LinMikle D. LedgerwoodCarolina MachadoMiku SodhiCaroline LangMi-Kyoung ChoCaroline Prot-BertoyeMilan ČižmanCaroline SchnakersMilan KolářCarolyn NessimMilan TomaCary PresantMilen DimitrovCasimiro Cabrera-AbreuMilena RajicCassandra ChaneyMilena Santric MilicevicCassie S. MitchellMilena ŚciskalskaCat KutayMilena VasicCătălina Iulia SăveanuMilica DeklevaCatarina GodinhoMilica M. BadžaCatarina MirandaMilind PadalkarCate ThomasMiljana JovandaricCaterina BonfiglioMilka DonchinCaterina Francesca GuidiMilon IslamCaterina SagnelliMiłosz CabanCatherine FreelandMiłosz LewandowskiCatherine N. KibirigeMilton Carlos Elías EspinosaCecilia ChanMin ChenCecilia EstradaMin LiCecilia Pegelow HalvorsenMin Sun KimCecilia PerinMinerva CruzCecilia VarjuMing ChenCecilie RøeMing Hsien TsaiCelia HardingMing-Chieh ShihCelia Ruiz-SánchezMing-Fong TsaiCelia SánchezMinghang WangCéline LoinardMing-Jui HungCem Cagri DonmezMing-Shu ChenCengiz AkbulutMingxing XieCésar CaraballoMingzhe JiangCesar Ivan Aviles GonzalezMinh Hieu NguyenChahnez Charfi TrikiMinh NguyenChaitanya DendeMin-Ho HanChaitanya GadepalliMin-Hui LiChamara V. SenaratnaMin-Seong HaChan Hee ParkMiraj Ahmed BhuiyanChanan ShaulMiranda DallyChander Singh DigwalMircea-Catalin FortofoiuChang Gue SonMireia LlauradoChang Won LeeMirela Baus LončarChang Yu HongMiren Altuna-AzkargortaChanghyun RohMiri KrupkinChang-Hyung LeeMiriam ElbrachtChangkun HuMiriam HaaksmaChang-Myung OhMiriam SaridChangwei WeiMirka SaarelaChangwon JeongMirko DuradoniChang-yun LinMirko ManchiaChangzhen WangMirna A. FawazChan-Young KwonMirona Palczewska-KomsaChao ZhangMiroslav BačaChaoling ChenMirza PojskicCharat ThongprayoonMishall Al-ZubaidieCharles AllenMishel Unar-MunguíaCharles JonassaintMister Gidion MaruCharles MoutonMitch ClarkCharles ScottMitchell DwyerCharli ErikssonMitsuru ShimizuCharlotte Kelley-JonesMitsuya YamakitaCharlotte PoolerMi-Yeon SongCharlotte RoyeenMi-Young ChoiCharlotte SteenblockMladen AndjićCharoula NikolaouMladena Lalic-PopovicChasity BrimeyerMobashir MohammadChatchaporn UraipongModesti Martina NicoleChathurangi PathiravasanMohab M. IbrahimChayoung KimMohamed M. DarwishChen CaiMohamed MahfouzChen YangMohamed MohanyChenguang DuMohamed R. AbonazelChen-Hsun WengMohamed S. EliwaChenlu GaoMohamed SalahudeenChen-Yi SongMohammad AlanafeaChenyu SunMohammad AlmseidinCheryl RoumenMohammad AltamimiChhaya PatelMohammad Al-WardatChi Kin KwanMohammad AshfaqChia-Chi WangMohammad AzadChia-Hong YengMohammad Belayet HossainChiaki ToidaMohammad Fahad UllahChiao Xin LimMohammad Farooq UmerChiara OppiMohammad Ghazi ShahnawazChiara SorberaMohammad H. Nadimi-ShahrakiChia-Yu ChenMohammad Hossein KhosraviChien-Chang ChenMohammad HosseiniChien-Hung LeeMohammad Jamshed SiddiquiChien-Te FanMohammad KhoveyniChifa HungMohammad Khursheed AlamChih-Chi ChenMohammad MehdizadeChih-Long LinMohammad Meraj MirzaChih-Ming LiangMohammad Mizanur RahmanChi-Hung LeeMohammad Mohiminul IslamChih-Wen WuMohammad RababaChi-Jane WangMohammad Reza MahmoudiChin-An WangMohammad ShboolChinedu Ogbonnia EgwuMohammad Yawar YakoobChing-Chung HsiaoMohammad ZamaniChing-Yi ChangMohammad ZulkifliChin-Hsien HsuMohammadjavad ZibaeenezhadChin-Hsuan LiuMohammadreza ShalbafanChintan TrivediMohammed A. Al-AbriChiranjibi SitaulaMohammed AlasawshChiu-Hung ChenMohammed AsadChi-Wen LungMohammed BazChi-Yu LeeMohammed Ejaz HussainChlebowska-Śmigiel AnnaMohammed F. H. BalahaChloe Meng JiangMohammed GrawishChng Saun FongMohammed H. MohammedChong Chen (Japan)Mohammed I. YacoubChong Chen (USA)Mohammed Oday EzzatChong-Chi ChiuMohammed R. S. SunoqrotChris GilletteMohammed RasheedChris JonesMohammed SayedChris LabakiMohammed Sultan Al-Ak’HaliChris PauluMohd Asyraf ZulkifleyChristian BarbatoMohd Hafiidz JaafarChristian KaczmarekMohd HanafiChristian LunghiMohd ImranChristian MesengeMohd Noor NorhayatiChristian NapoliMohd. Zulfaezal Che AzeminChristian SchachMohmed Isaqali KarobariChristian SchranzMohsen AminizadehChristian Velasco-GallegoMohsen KeshavarzChristian WeissMohsen ShahhosseiniChristian WernerMohsen SoltanifarChristiane BayerlMohsen YazdanianChristina CoughlanMoises Alejandro Alatorre-JimenezChristina MejersjöMoises Leon-JuarezChristina ScharfMoisés MarquinaChristine A. JamesMojdeh BanaeiChristine Lux‐BattistelliMojtaba KavianiChristine Manlai KwanMojtaba LotfalianyChristine Maria SchwarzMojtaba VaismoradiChristine RogersMolly C. NicodemusChristine San GiovanniMomcilo JankovicChristine StierMominur RahmanChristoforos NtantogianMona Abdelbaset Sadek AliChristoph DodtMona VintilaChristoph FaschingerMonica GulatiChristoph StallmannMonica L. RobinsonChristophe DomingosMonica LöfvanderChristophe FehlmannMónica Martínez-GómezChristophe Le TerrierMonica Pinilla-RoncancioChristopher Adair-ToteffMónica Ríos-SilvaChristopher AmalrajMonica SalernoChristopher J. DestacheMónica TeixeiraChristopher J. WalinskiMonika DąbrowskaChristopher Keith HaddockMonika E. RacChristopher KobierzyckiMonika FranczukChristopher KotarskyMonika KlimekChristopher L. AtkinsonMonika Klinkhammer-SchalkeChristopher Paul EcksteinMonika Michalek-ZrabkowskaChristopher R. GustafsonMonika Morawska-KochmanChristos K. YiannakopoulosMonika PechhackerChristos MikropoulosMonika RuszałaChristos PaizisMonika WernetChristos SikarasMontree TungjaiChristos TsagkarisMontserrat Diéguez-PérezChrysi KoliakiMoo K. ChungChuen-Fu LinMoon Fai ChanChun GaoMor Nadia Lurie-WeinbergerChun Hung ChuMoraitou DespinaChun-Che HuangMoran BenistyChung Y. HsuMoran PollackChung Yi LiMoreno BaruffiniChung-Feng LiuMoreno ZanardoChung-Fu HuangMorten HesseChung-Hsin WuMostafa GoudaChung-Kan PengMotolani OgunsanyaChun-Gu ChengMoudatsou MariaChung-Ying LinMourouzis PetrosChunki FongMoushumi RoyChunmei CaoMoustafa NasrallaChyi-In WuMo-Yeol KangChyntia JasirwanMriganka SinghCiaran StanleyMuawuz IjazCiavoi GabrielaMubarak S. KarsanyCid André Fidelis De Paula GomesMudassar Iqbal ArainCid Marcos Gonçalves AndradeMuddassar SarfrazCihan KayaMuhamad Hilmil Muchtar Aditya PradanaCindy A. PrinsMuhammad A. RahmanCintia Chamorro-PetronacciMuhammad Adeel AhmedCinzia RienzoMuhammad AkramCiro Di NunzioMuhammad Aksam IftikharCiro EspositoMuhammad Amir KhanCiro José Jardim De FigueiredoMuhammad AsimClare McKeaveneyMuhammad AttiqueClarissa FerrariMuhammad Azeen AshrafClaro BichoMuhammad Azher BhattiClaude DussartMuhammad BilalClaudia CameddaMuhammad FarooqClaudia LermaMuhammad Hammad ButtClaudia LoretiMuhammad Heikal IsmailClaudia MehedintuMuhammad ImranClaudia PisanuMuhammad Imran RashidClaudia QuaresmaMuhammad IrfanCláudia Regina Furquim De AndradeMuhammad IrshadClaudia TodaroMuhammad N ZahidClaudimar Pereira Da VeigaMuhammad N. MahmoodClaudina NogueiraMuhammad NoumanCláudio Daniel CerdeiraMuhammad OmairClaudio De LuciaMuhammad Phetrus JohanClaudio FeoMuhammad RasoolClaudio VelatiMuhammad RiazClaudiu MargineanMuhammad ShafiqClaudiu MorgovanMuhammad Sheikh SadiClayton SmithMuhammad TufailClementine LabroscianoMuhammad Umar ChaudhryClifton AddisonMuhammad ZeeshanClifton HolmesMuhammed SaeedClive FriedmanMuhammed Sedat SakatColin DrummondMuhammed UnlersenColin K. DrummondMuhammed Yunus BektayColin MulliganMuhammet Fatih AslanColleen M. GallagherMukhtar AnsariConcetta Di NoraMuna BarakatConcetta PirontiMunawar NaylaConcetta TomainoMunawir MunawirConcha AntónMurad HossainConsolato M. SergiMurad RassamConstantin PotagasMurat EyubogluConstantine KotropoulosMurat Şakir EkşiConstantino Carlos Reyes-AldasoroMusheer AljaberiConstantinos KoutsojannisMustafa CesurConxita MestresMustafa Said YıldızCorina Aurelia ZugravuMustapha KrimCorina GüthlinMuthu SathishCorina IoanășMuyiwa OladosunCorina VasileMuzamil Basha SyedCorinne SquireMylène DuivonCornelia MirceaMylynda Beryl MassartCorrado CiattiMyongwon SeoCorrado ColapricoMyoungjin KwonCorrado Lo StortoMyra S. SchmadererCorrado TagliatiMyung-Chul KimCorrine Warren RuktanonchaiN. Rich NguyenCosmin CituNada MarićCostantina ProtaNadav RappoportCostantino CiallellaNadia DepetrisCostas S. ConstantinouNadia NajafiCourage MlamboNadinne RomanCovadonga Díez-SanmartínNaeem HayatCraig A. H. RichardNaeem JanCraig Stephen WebsterNaemeh NikvarzCraig UlrichNagehan AslanCreed JonesNagisa SugayaCricket MeehanNagwa Ali SabriCrismita DmelloNa-hyeon (Hannah) KoCristian DelceaNailya DelellisCristian FrancavillaNajat YahiaCristian LieneckNaji AlqahtaniCristián OyanadelNajm AlshahraniCristian RotariuNana Amoah-RameyCristian TrambitasNana Kwadwo BiritwumCristiana GambelungheNancy BylCristiane Yumi Koga-ItoNancy DoetzelCristina B. NevesNancy HelouCristina BaixinhoNancy McNaughtonCristina De DiosNancy TofilCristina Espinosa-SempereNandaraj TayeCristina FrangeNan-Ping YangCristina GavriloviciNaoki IkegayaCristina GiannattasioNaoki SudoCristina GrippaudoNaoko KatsuradaCristina M. Beltran ArocaNarasimha Rao PillalamarriCristina Manuela DrăgoiNarayanankutty ArunaksharanCristina Mendoza-HolgadoNarendar DudhipalaCristina MondelloNarendra Babu KondapelliCristina MonteiroNaresh KojuCristina Moreno-MuletNarimasa KumagaiCristina NunesNarrendar RaviChandranCristina Rivera-PicónNaseer AhmedCristina SecosanNasire UlucCristina TeixeiraNasser AlamiriCristina-Mihaela LăcătușuNatalia Calvo AyusoCristo M. Marrero-GonzálezNatalia Cezon-SerranoCrystal MarrugantiNatalia DanekCynthia BlantonNatalia DowgiałłoCynthia L. BodkinNatalia GrzebiszCynthia WhissellNatalia Hernández-SeguraCyril FersingNatalia Kaźmierczak-WojtaśCyrus MotamedNatalia Maria SerwinDa Yang TanNatália MartinsDafna HalperinNatalia RakislovaDahang YuNatalie BrownDahlia M. KenawyNataliia TeresDai PuNatallia DubashynskayaDaicong DaNataša RančićDaiji NagayamaNathan Kumasenu MensahDaisuke KikuchiNathanael OjongDaisuke MachidaNathaniel McQuayDaisuke MatsushitaNathaniel T. BerryDaisuke TakayanagiNato DarchiaDaisuke WatanabeNaveen ThackerDaKysha MooreNavid AbedpoorDale KirbyNavid GhasemiDalea M. BukharyNavid Jubaer AyonDalia AlMaghaslahNavid OmidifarDalia El KhouryNavin Kumar DevarajDaly AvendanoNawab Muhammad Faseeh QureshiDamian CzarneckiNebojša D. ZdravkovićDamian PawlikNefyn WilliamsDamiano PatronoNeha DesaiDamiano PerriNejc UmekDan JiangNemesio Villa-RuanoDan RomerNenad KoropanovskiDan VoiculescuNenggang XieDana A. BrownNerea Gutiérrez-FernándezDana AlbonNesrein M. HashemDana BadauNeves-Amado JoãoDana Balas-TimarNgoc Dung BuiDana BradfordNgoc Thang BuiDana LawrenceNguyen Minh DucDanail PavlovNguyen Vinh KhuongDan-Alexandru SzaboNi ZhangDanfeng SunNiamh CoffeyDaniel CrabtreeNianye ZhengDaniel Das Virgens ChagasNiccolò PersianiDaniel Demétrio Faustino-SilvaNicholas D. LawsonDaniel DziobNicholas DopkinsDaniel FuentesNicholas GrubicDaniel García IglesiasNicholas K. SchiltzDaniel Josef LindeggerNicholas ReedDaniel KellyNick Platon SachinisDaniel López-LópezNicola Alberto ValenteDaniel Lopez-MaloNicola Di FazioDaniel M. KirganNicola GentiliDaniel Patiño-GarcíaNicola LovecchioDaniel Romaus-SanjurjoNicola MagnavitaDaniel Sanz-MartínNicola MarottaDaniel SchulzeNicola MucciDaniel ŚlęzakNicola NanteDaniel SteinmannNicola PalenaDaniela Carmen AbabeiNicola SheeranDaniela CavacoNicola TambascoDaniela De VenutoNicola TartagliaDaniela DibelloNicolae GicaDaniela HaluzaNicolae PopaDaniela LeccaNicolas BergerDaniela MariNicolas BraultDaniela Opris-BelinskiNicolas PallikarakisDaniela ViscontiNicoletta De SantisDaniela VrinceanuNicolina CapoluongoDaniele Della LattaNida AslamDaniele GiansantiNidia N. GómezDaniele NucciNiels Damkjær OlesenDaniele Ugo TariNien Mu ChiuDaniele UrsoNigel D. RobbDanielle FurgesonNika PavlovićDanielle R. EvangelistaNikita Khromov BorisovDanijela Ristić-MedićNiklas CederströmDanijela TrifunovicNikola StefanovićDanilo ContieroNikolaos EfstathiouDan-Marius DobreaNikolaos KatzilakisDan-Mircea OlinicNikolaos KotsanosDanny OttoNikolaos LaliotisDanúbia Da Cunha De Sá-CaputoNikolaos MachairasDanuta Lietz-KijakNikolaos SoldatosDanuta UmiastowskaNikolaos TsetsosDanya MuilwijkNikolaos ZarasDanyelle GreeneNikoleta PrintzaDara JamesNikos ViazisDardan KlimentaNikša DulčićDarin ZertiNilufeur McKayDarinka AckovaNina Brkić-JovanovićDario BabicNina LansburyDario LipariNina TumosaDariusz DrążkowskiNir BarDariusz KałkaNirmal Kumar MohakudDariusz MoslerNiruwan TurnbullDariusz RydzNirvana PopescuDariusz Wojciech MazurkiewiczNisansala AbeyrathnaDarko ModunNishant Raj KapoorDarryl PlecasNitin GoyalDarshan KelleyNitin KhandelwalDastan BamwesigyeNitzan Peri-RotemDavid Baeza MoyanoNnennaya U. OparaDavid BarneyNobuari TakakuraDavid BeckNobuhiro AriyoshiDavid BentleyNoelia González-GálvezDavid CatelaNoemi GiannettaDavid CingranelliNoor Zaman JhanjhiDavid ConnerNopadon KronprasertDavid ElyNora RepoDavid González-MartínNora Suleiman-MartosDavid Hernández-GuillénNorah AlmusharrafDavid J. ChesireNoriaki IwahashiDavid Llaneza-SuarezNorihiko NaritaDavid LucasNorintan Ab-MuratDavid MadiganNorio OtaniDavid Manzano-SánchezNorio YamamotoDavid MartinNorma G. Padilla-EguiluzDavid MoleroNoureddin Nakhostin AnsariDavid Moreno MolinaNoureddine LakouariDavid MusokeNuno CrespoDavid NussbaumNuno LavadoDavid OliverNuno MoraisDavid PakelianiNunzio CirulliDavid Ribas PérezNupur DasDavid RosenthalNurhanis Syazni RoslanDavid Sánchez-MolinaNurul Hashimah Ahamed Hassain MalimDavid Sancho-CantúsOak-Hee ParkDavid Sarabia-JácomeOana AlmasanDavid Valle-CruzOana Dragos-PinzaruDavid WrightOctavian AndronicDavide BorroniOgul Ersin UnerDavide CarusoOhad RonenDavide CostaOkechukwu ChukwudehDavide NegroniOkyaz EminagaDavinia VicenteOlaf GefellerDawid KoźleniaOleg LeonovDawid MajcherekOlfat SalemDawid StormanOlga Czerwińska-LedwigDawid SzwedowskiOlga De Cos GuerraDawson ChurchOlga GutkowskaDayun YanOlga JensenDe DasguptaOlga L. VoroninaDe Rosal Ignatius Moses SetiadiOlga ValentimDean David Ad-ElOliver GröneDebbie FaulknerOliver HenkeDebbie LingOliver J. OttDebopam SamantaOliver Mendoza-CanoDebora ColangeloOliver Ramos ÁlvarezDeborah H. AllenOlivia KungumaDeborah RundOlivier FogelDeborah Witt ShermanOliwia Gawlik-KotelnickaDebra Wetcher-HendricksOlmos GómezDeepa GuptaOluwagbemiga DadematthewsDeepak KumarOm Prakash ChoudharyDeependra Kumar RaiOmar AlzubiDejan BudimirovicOmar El HibaDelfín Ortega-SánchezOmar JanehDelia HorhatÖmer AlkanDelia Mirela TitOmer UnsalDemetrio LozanoOmid MirmosayyebDemóstenes Zegarra RodríguezOmkara Swami MuddinetiDenis GallotOmneya AttallahDenis LazarenkoOnofrei PavelDenise PergolizziOPhir NaveDenisse ParraOrhan KoçakDennis WaskoOriana SimonettiDeodato AssanelliOriol MitjàDeolinda M. L. Dias RasteiroOrly SaridDerek P. EvansOrsolya VargaDerek SmolenskiOsamah Al RugaieDermot BurkeOscar CrisafulliDer-Shin KeÓscar ZurriagaDesirée Mena-TudelaOsnat BashkinDessy RachmawatiOsvaldo GervasiDesu ChenOtto LamDevi MohanOtto RuokolainenDhananjay YadavOurania TremmaDhara SharmaOussama SaidiDhaval PauÖzgü İnalDhuha WazqarÖzgün ÜnalDi Wuözgür BostancıDian Candra Rini NovitasariOzgur Koray SahingozDiana Angelika OlszewskaP. T. Martín De La VegaDiana Angelika SteppanPablo A. Cantero-GarlitoDiana CelebańskaPablo Ezequiel Flores KanterDiana KwokPablo FaríasDiana L. SpeelmanPablo Hernandez-LucasDiana MartinsPablo RuisotoDiana Rodríguez-HurtadoPablo Valdés-BadillaDiane ClarkPak Hin Hinson CheungDibya PandaPalanikumar BalasundaramDidar UcuncuogluPaloma Echevarría-PérezDidier RiethmullerPamayla DarbyshireDidik Setyo HeriyantoPamela BeachDiego Giulliano Destro ChristofaroPamela Di GiovanniDiego Gomez-BayaPanagiota G. PietriDiego H. CáceresPanagiota GiakkoupiDiego Marqués-JiménezPanagiotis F. LiaparinosDiego Soto GarcíaPanagiotis G. HalvatsiotisDiego TlapaPanagiotis GkriliasDiego VasconcellosPandurang KolekarDifang HuangPang-Yun ChouDilaver TengilimogluPaniz JasbiDilbag SinghPankaj ChauhanDillon MintoffPanpan ZhouDima SabbahPaola FratiDimitra Rafailia BakaloudiPaola GargiuloDimitrios A. VrachatisPaola QuaresimaDimitrios KasselimisPaolo Albino FerrariDimitrios PanagopoulosPaolo BaldoDimitrios PapagiannisPaolo CanepaDimitrios StamovlasisPaolo CapparèDimitrios TsarapatsanisPaolo CiampaliniDimitrios VlachopoulosPaolo ContieroDimitris ZavrasPaolo Emilio SantoroDina Di GiacomoPaolo GasparellaDina IzenstarkPaolo MartellettiDina M. El-SherifPaolo MeneguzzoDinesh K. ThekkinkattilPaolo PascoloDinesh MittalPaolo PellegrinoDinesh RokayaPaolo PeregoDinithi PeirisParastoo FarniaDino ŽupanovićParisa K. KargaranDiogo Luís MarquesParisa Moll-KhosrawiDiogo Thimoteo Da CunhaParisi AttilioDiogo VidalParshva PatelDipto SinhaParvaiz Ahmad NaikDivya BeriPascal JungbluthDivya GopinathPasquale AuricchioDivya NairPasquale Fabio ProvenzanoDivya SinhaPasquale InnominatoDjordje MedarevicPasquale LoiudiceDmitrii ShekPasquale LongobardiDmitriy ShcherbakovPasquale PisapiaDoga KuruogluPasquale StefanizziDolores Rando-CuetoPasqualina BuonoDomen PlutPatorn PiromchaiDomenic MarbaniangPatricia C. García-SuárezDomenico CiavarellaPatricia Correa-GhisaysDomenico DalessandriPatricia DiogoDomenico MelidoroPatricia GarnicaDominic AugustinePatricia HuelinDominik DłuskiPatricia Otero OteroDominik JańczewskiPatrícia PiresDominik OlejniczakPatricia SauthoffDominik RadzkiPatricia VermeerschDominika KalankovaPatricio Ramírez-CorreaDomitille CallonPatricio SánchezDomonkos VargaPatrick A. PalmieriDonatas AustysPatrick HearingDonatella CiarapicaPatrick PalmieriDonatella Di CorradoPatrik DridDonatella VerbanacPatrycja KleczkowskaDone StojanovPatrycja Romaniszyn-KaniaDong Keon YonPatrycja ZurzyckaDong WangPatryk RzońcaDong Wook ShinPatryk TarkaDonghwan ParkPau Redón LurbeDongming ChenPaul AjahDongwei ZhangPaul BatchelorDongwoo LimPaul DonahueDonovan McGrowderPaul E. EngelhardtDor AbrahamsonPaul Eze EmeDora-Luz FloresPaul GissenDorin IonescuPaul HodkinsonDoris RusicPaul J. AllisonDorit GamusPaul KindDorota CzyżowskaPaul OakleyDorota DiakowskaPaul S. AmieuxDorota KleszczewskaPaul S. HafenDorota LasotaPaula BeneveneDorota OzgaPaula BoaventuraDorota RaczkiewiczPaula ChavesDougho ParkPaula Fernández GarcíaDouglas HanesPaula Fernández-PiresDouglas WrenPaula FerreiraDo-Youn LeePaula GomezDragan HrnčićPaula LagoDrago JelovacPaula Peral GómezDraško PavlovićPaula Rodriguez-ModroñoDrazen CularPaula VianaDulce Romero-AyusoPaulina AdamskaDumitru CiolacPaulina KrawiecDunja DegmečićPaulina NowaczykDurga Kalyani PaturiPaulina PobleteDušan KlosPaulina PolkoDushyant DamaniaPauline HarperDuygu AğagündüzPaulo AlvesDuyu NiePaulo MartinsDyana OdehPavel BachmannDylan M. JohnsonPavle BanovićDyson T. WakePawan Kumar RaghavDzati Athiar Bt RamliPaweł ChmuraDziedzic ArkadiuszPaweł GaćDzifa DordunooPaweł LarionowE. Begoña Garcia-NavarroPaweł LinekE. David G. McIntoshPaweł PakoszE. Miranda ReiterPaweł PrędkiewiczEaswaramoorthy RangaswamyPaweł WańkowiczEbrahim Abbasi OshaghiPaweł WittEbrahim BanitalebiPayam Safavi‐NaeiniEbrahim NajafzadehPayam ShojaeiEdel EnnisPearl Pei LiuEdgar CambazaPedro A. Martin CervantesEdina EszenyiPedro BarataEdinson Dante Meregildo RodriguezPedro C. Santana-MancillaEdisson-Mauricio Pacheco-QuitoPedro CandeiasEdit XhajankaPedro Espada-SantosEdita FinaPedro FerreiraEdita FinoPedro FonsecaEdith ClarosPedro Juárez RodríguezEdith ClassenPedro L. Pancorbo HidalgoEdith Lillian Roth GjevjonPedro LucasEdna AcostaPedro ManonellesEdna PatatanianPedro Manuel Rodríguez-MuñozEdoardo BraunerPedro Mendes LoureiroEdoardo MonfriniPedro Miguel Alves Ribeiro CorreiaEdoardo PireraPedro O. RosselEdoardo RellaPedro Paulo Menezes ScariotEdsaul Emilio Perez-GuerreroPedro PiñeroEdson SilvaPedro SobreiroEduard BaladiaPedro Valdivia-MoralEduard GuaschPedzisai MakoniEduardo CostaPeera WongupparajEduardo Gutiérrez-AbejónPeggy KayEduardo Hernández-PadillaPei YuangEduardo José Fernández RodríguezPeichao ChenEdward ChiyakaPei-Fen YenEdward F. FitzgeraldPeiman GhasemiEdward HofferPeivand BastaniEdward J. PavlikPekka TarkkilaEdward LebakaPenelope June WellerEdward MissanjoPeng ChenEdyta MądryPeng GaoEeva AuvinenPeng HuEfraín Santiago-RodríguezPeng ZhangEggi ArguniPere Lavega-BurguésEgidia G. MiftodePerman GochyyevEgidio CelentanoPernille HolmagerEgoitz AstigarragaPetca Razvan-CosminEhsan AsadollahiPeter BrödeEhsanul Hoque ApuPéter CsécseiEhtesham HassanPeter GänglerEirini GrapsaPeter GoodwinEirini OrovouPeter KondrlaEitan FrachtenbergPeter KorstenEitaro KodaniPeter Lloyd-SherlockEjemai EboreimePeter Modupi MphekgwanaEkaterina BaryshnikovaPeter OttEkaterina GeorgievaPeter SchemmerEl Hadji M. DioumPeter W. ChoateElahe HosseiniPeter WakholiElena A. KorolevaPetr SedláčekElena CampelloPetra CzarniakElena CommodariPetra PejićElena De-La-Barrera-ArandaPetre BrezeanuElena Di PierroPetre ChipuriciElena DruicaPetronila OlivaElena Escolano-PérezPetros DinasElena GrimacciaPey Yee LeeElena Mainer PardosPhatthranit PhattharapornjaroenElena MarchioriPhil AyersElena Martinez-SanzPhil MaudeElena MunariniPhilip LiElena Muñoz-GómezPhilipp SischkaElena Pinero-PintoPhilippe CollotteElena RezusPhilippe GuillaumeElena RizaPhu HoangElena SîrbuPhyllis GasparElena TarcaPhyllis GendlerElena V. IndukaevaPia Lopez-JornetElena WeitzelPierfrancesco GrimaEleni GavriilakiPiergiusto VitulliEleni TolmaPiero Vincenzo LippolisEleonora NuceraPierpaolo Di LorenzoEleonora PintoPierre Fernand NeuenschwanderEleonora PretiPietro CacialliEleonora SantosPietro FerraraEleonora TopinoPietro MazzeoEleonora VatamanPietro MorroneEliã Pinheiro BotelhoPietro ScicchitanoEliane CastroPil Young JungElias MpofuPilar Aparicio-ChuecaElif Seda Selamet TierneyPilar Marques-SanchezElif Uygur-KucukseymenPilar Munuera GómezElin KjellePilar Pérez-RosElinor HarrisonPilar VílchezElio BorgonoviPinar KorkmazElio VenturiniPinchas HalpernElisa CabanaPinelopi ArvanitiElisa ReitanoPing LuElisa SconfienzaPing SongElisabet Fernández-GómezPing WangElisabet Roca-MillanPing ZouElisabeth BondessonPing-Fing ChiuElisabetta BertolPin-Kuei FuElisabetta Di FelicePinto-Almazán RodolfoElise Arsenault KnudsenPinuccia FavianaElisete DiogoPiotr AschenbrennerElisete MourãoPiotr BeraEliya LevingerPiotr BiałasElizabeth C. PenickPiotr FormanowiczElizabeth DodsonPiotr GabryelElizabeth Morales-MorenoPiotr GałeckiElizabeth SchieberPiotr GronekElke DoberentzPiotr KarniejElke KniselPiotr KornetaEllen F. MoslethPiotr LeszczyńskiEllen FunkhouserPiotr MalaraElliot LassonPiotr MatłoszElmedin BajricPiotr MorasiewiczEloísa Fernández-OrdóñezPiotr PoznanskiElpidio Maria GarzilloPiotr RatajczakElsa PegadoPiotr RegulskiElsa VitalePiotr RolaÉlvio Rúbio GouveiaPiotr SkrzypczakElza Maria Morais FonsecaPiotr TutkaElżbieta MillerPiotr TworekElżbieta PoniewierkaPiotr WychowańskiEman Abu-GharbiehPiyameth DilokthornsakulEmanuela TurillazziPiyush GuptaEmanuele BarabinoPoh Foong LeeEmanuele CassioliPooneh SalariEmanuele RizzoPoppy WatsonEmeline FresnelPosen LeeEmiel F. M. WoutersPoulami DattaEmilia AlvesPrabal BaruaEmilia CostaPrabhat K. TalukdarEmilia PapakonstantinouPrachi ChavanEmilien JeannotPradeep Kumar DasEmilio GrecoPradipta BhaktaEmily RobertsPrajakta MasurkarEmine İkbalPrakash SahEmma CarlinPramesh SinghEmma MontellaPramod GaurEmmanouil G. SpanakisPrasart NuangchalermEmmanouil GiorgakisPrasenjit SahaEmmanuel FrimpongPrashanth Thevkar NageshEmmanuel J. FavaloroPratima KumariEmyr BenbowPratiti BhadraEnder KazazogluPraval KhanalEndrin KoniPraveen Kumar BhartiEngin ArikPraveen Kumar NeeliEngy ElekhnawyPraveen Kumar Reddy MaddikuntaEnkelejda KasneciPrawej AnsariEnquan XuPrawin KumarEnri NakayamaPrecious N. SangoEnrico ApaPrerna VarmaEnrico BrunettiPritam BoseEnrico CapobiancoPrzemysław DomaszewskiEnrico Fuini PugginaPrzemyslaw Falkowski-GilskiEnrico GiordanPrzemyslaw PlonkaEnrico Maria StaderiniPrzemysław Robert NowakowskiEnrico OddonePrzemyslaw WolakEnrique Meléndez-HeviaPrzemysław ŻuratyńskiEnrique Sáez-AlvarezPu HaoEnrique Urrea-MendozaPuhong ZhangEnzo Maria VingoloPurva V. SharmaEpameinondas GousopoulosQaiser RiazEraldo OcchettaQi GaoErfan Babaee TirkolaeeQi JinErginbay UgurluQi ZhangEric MartinQiang GongEric OpokuQianqian PanEric PeeplesQianyi FuEric SonaQihui ChenErich KastenQimin HaiErick Andres Perez AldayQin FuErick Martínez HerreraQinaat HussainErick Sierra-CamposQing LiErika Aparecida SilveiraQing YeErika Bevilaqua RangelQingming FangErika BonacciQinzhe WangErika JoosQiqi ChenErika ZelkoQiuping LiErin MaughanQuan-Hoang VuongErin MorganQuanjun LyuErsan ArslanQueirós CarlaErshuai ZhangQuentin D. ReadErsin KayahanQun WangErtuğrul ŞahinQun WeiEsma ÖzkanQuy KhucEsperanza Begoña García NavarroR. AnandEsperanza Clares-ClaresRabia Sannam KhanEssam Ahmed Mohammed Al MoraissiRabiu Muazu MusaEssam SolimanRachel ChildersEsteban Obrero-GaitánRachel JewkesEsteban SepúlvedaRachel KafriEstefania NuñezRachel SilvermanEstela BlancoRachel WhitsedEsther Medrano-SánchezRachele MarianiEstíbaliz JiménezRachelle MendozaEudald FelipRachid Ait AddiEugen Romulus LontisRachida DalouhEugenia VlachouRadoslav BeňušEulicio De Oliveira Lobo-JuniorRadoslava KralevaEun Jeong HwangRadu BotgrosEun Kyoung ChungRadu ComesEun Young ParkRa'ed Masa'dehEunice CarrilhoRaeesa GupteEunju ChoiRafael Camacho-CarranzaEunjung HyunRafael Coutinho Mello MachadoEun-Jung KimRafael Escamilla-NunezEunyoung HanRafael Galán-ArribasEun-Young ParkRafael JodarEva AusóRafael OliveiraEva Ausó MonrealRafael Salido-VallejoEva BorrasRafael Villalba-MontoroEva Ekvall HanssonRafaela VasiliadouEva Maria Navarrete-MunozRafaella Pessoa MoreiraEva María Sosa-PalancaRafal BuldakEva PodovšovnikRafał GerymskiEva TsaousidouRafal KaminskiEva TvrdaRafał SkowronekEva-Maria RisoRafał StemplewskiEvangelia AntoniouRafeeya ShamsEvangelia KoukakiRaffaele BaioEvangelos SymeonidisRaffaele De CaroEvelin WitrukRaffaella CancelloEverson MeirelesRaffaello PellegrinoEvgenia GkintoniRaghavan GopalakrishnanEvgeniy CherepovRaghu GandhiEvgeny R. BojkoRaghunadharao DigumartiEvika KaramagioliRaghunathan KrishankumarEvridiki KabaRahim AbdurEwa ChlebuśRahimeh RouhiEwa Czarniecka-SkubinaRahul GuptaEwa Gibuła-TarłowskaRahul SharmaEwa GrudzińskaRail M. ShamionovEwa LangeRaili KauppinenEwa LatourRaimondo PianaEwa NowakRainer LeonhartEwa Otto-BuczkowskaRainer ReileEwa Puszczalowska -LizisRaja Ariffin Raja GhazillaEwa RaczkowskaRajaguru VasukiEwa RodakowskaRajani SinghEwa SawickaRajasekhara NerimetlaEwa SobolewskaRajat Emanuel SinghEwa TomaszewskaRajeev DwivediEwelina ChawłowskaRajendra NathEwelina DziurkowskaRajesh ChaudharyEwelina NojszewskaRajiv JanardhananEziuche Amadike UgboguRajiv SinglaFabian SchrumpfRajkumar SahaniFabiana LucàRajneesh KumarFabio D'AndreagiovanniRaju AnandFabio Del DucaRakesh KhilwaniFábio Juner LanferdiniRakeshkumar B. PatelFabio MaggioRalph PooleFabio PellegrinoRaluca DumacheFabio SaliceRaluca IsacFabio SantacaterinaRam Kinker MishraFabio TimeusRam RamamurthiFabrice DarcheRam SagarFabrício Ramos Silvestre PereiraRam SharmaFabrizio CedroneRamachandran PrakasamFabrizio ConsortiRamendra Pati PandeyFabrizio D’AscenzoRamesh ChandrababuFabrizio GuerraRamesh Kumar MuralimanoharFabrizio MartoraRamey MooreFabrizio TuroldoRami N. Al-RohilFahad A. AlhumaydhiRamit Maoz-SegalFahad AsmiRamune JacobsenFahad KhanRan AnFahimeh RanjbarRandi VitaFaisal AlghayadhRanjan K. MohapatraFaisal F. HakeemRanjith KamityFaizah Safina BakrinRanko ŠkrbićFakhredin SabaRaphael CiumanFan WuRaquel EchavarriaFangyao TangRaquel Lara MorenoFanny Baran-MarszakRaquel Leirós-RodríguezFarah MulyasariRaquel Lozano BlascoFarida SaleemRaquel PradaFaris Shuleih Mohammad AlshammariRaquel Sánchez-RecioFarnaz Ghazi-NezamiRaquel Simões De AlmeidaFarooq AhmedRasa BarkauskienėFaruk Fonthal RicoRasa JankauskieneFarzad Taghizadeh-HesaryRasa MladenovicFatemeh DabbaghRashed NoorFatemeh KhatamiRasoul MirzaeiFatima AroojRatko PrstačićFatima FalcaoRaul Alberto Carrilho CordeiroFátima RoqueRaul AntunesFatma LestariRaúl Cuauhtémoc Baptista RosasFatwa DewiRaúl Díaz-MolinaFauna HerawatiRaúl DomínguezFaustin Armel Etindele SossoRavali GourishettiFaustine WilliamsRavi Philip RajkumarFausto AssandriRavi Shankar ReddyFawaz AlharbiRavil MuhamedyevFaye TaxmanRavindra Kumar JenaFazal MuhammadRay W. BasrowiFederica FogacciRaymond J. WallsFederica GalimbertiRaysa RochaFederica GalliRayssa Ferreira ZanattaFederica IntorreRay-Yu YangFederica MurmuraRazia AdamFederica TamburellaRazvan BoiangiuFederica VeroneseRăzvan George RahotăFederico Abate DagaRebeca LorcaFederico BiancoRebecca ErschensFederico D’AntoniRebecca GordonFederico Maria RubinoRebecca HubbardFederico MastroleoRecai ErgunFederico PennestrìRegina Ferreira AlvesFederico RoggioReima SuomiFedor DuzhinReinhard LängerFei LuoReinhard StrametzFeifei WangReinhold FueggerFelice BedfordRémi EsclassanFelipa De Mello-SampayoRemigijus BubnysFelipe J. AidarRena KostiFelipe José AidarRenata ChalasFelipe Ojeda PérezRenata GržićFelix Jimenez-RondanRenata PecoticFélix Marcos-TejedorRenata PiotrkowskaFelix Martin WernerRenata Puppin ZandonadiFeng ChangRenata VrsalovicFeng YuanRenata Wolfshaut-WolakFeng-Hua SunRenata WoźniackaFengqing ChaoRenata ZandonadiFengyu LiRenato HeidorFerdinando AgrestaRenato LeoneFerdinando FranzoniRene CorteseFerdinando RomanoRené HägerlingFereydoon LaalRenee CareyFernanda GentilRen-Fang ChaoFernanda TonelliRenzo ZanottiFernando AlacidReparata Rosa Di PrinzioFernando Augusto Ugusto Mardiros HerbellaReto BaertschigerFernando BarragánReza MazaheriFernando OlivaresReza MirshahiFernando Rubén García HernándezReza SaatchiFernando Salazar-ArrietaRiadul IslamFernando Suparregui DiasRicardo A. Cartes‐VelásquezFerruccio ConteRicardo Bernardez-VilaboaFeten Zar KalaiRicardo ChalmetaFilip GórskiRicardo FonsecaFilip JovanovicRicardo IbañezFilipa SousaRicardo Jorge Dinis-OliveiraFilipe FidalgoRicardo LoureiroFilipe MachadoRicardo MalheiroFilipe Paiva-SantosRicardo SilvaFilipe PrazeresRicardo VardascaFilippo ConfalonieriRiccardo MastroianniFilippo Manelli (ASST-Valcamonica)Riccardo MonterubbianesiFilippo Maselli (Sapienza University of Rome)Riccardo PascuzzoFilippo RapisardaRich GormanFiona CostaRichard David HaywardFiona EcarnotRichard E. HeymanFiorella Pia SalvatoreRichard F. IttenbachFiorenzo LupiRichard G. TrohmanFiras KobeissyRichard H. ParrishFirat AkcaRichard John Bruce FrancisFirooz SalamiRichard KahnFiroz AkhterRichard L. WolfelFitri OctavianaRichard M. PinoFlavio BertiniRichard ScepuraFlavio MilanaRichard Yudi HidaFlemming AndersenRiitta TurjamaaFlor H PujolRiku KawasakiFlora N. BalievaRim BourgiFlorenc DemroziRini Devijanti RidwanFlorence M. WeierbachRintu ThomasFlorence Mei Fung WongRiquelme Marín AntonioFlorent CarsuzaaRishi Man ChughFlorian FischerRishi PalFlorian KipfmuellerRita Alexandra Santos-RochaFlorian SchachingerRita DeeringFlorin GirbaciaRita KissForoud ShahbaziRita PavasiniFortunato FernandaRita SanctisFoteini ChristidiRitesh ChimoriyaFotios KirvassilisRitsuko Kimata PoohFrances Mary JohnsonRitthideach YorsaengFrancesca BorzìRittika ShamsuddinFrancesca CarissimiRobbert HuijsmanFrancesca CattoniRobert Alan SloanFrancesca CecchiRobert AlexanderFrancesca De FeliceRobert AncuceanuFrancesca DiolaiutiRobert B. SlocumFrancesca GiovannenzeRobert BoydFrancesca GuidaRobert BransfieldFrancesca LatinoRobert BurdettFrancesca NegroRobert D. RoghairFrancesca PisanoRobert DonatelliFrancesca Romana RipaniRobert E. LarsonFrancesca RossiRobert E. PykeFrancesco AgostiniRobert Emmett KellyFrancesco BaccelliRobert FlisiakFrancesco BarberoRobert J. SternbergFrancesco BellinatoRobert KarpińskiFrancesco CastagnettiRobert Konrad SzymczakFrancesco CollivignarelliRobert Philipp KosilekFrancesco CoreaRobert PodlasekFrancesco De MiccoRobert PrillFrancesco Di BelloRobert SusłoFrancesco FerriniRobert TragerFrancesco FortarezzaRoberta E. EmetuFrancesco GrandeRoberta MonzaniFrancesco LuparielloRoberta ZupoFrancesco MaffìaRobertas DamaševičiusFrancesco MassoniRoberto Abeldaño ZuñigaFrancesco MeaniRoberto BellucciFrancesco PorcelliRoberto BenoniFrancesco SegalaRoberto FornaraFrancesco SessaRoberto GeladoFrancesco StiloRoberto Giovanni Ramírez-ChavarríaFrancesco TommasiRoberto Oliveira DantasFrancesco VenturaRoberto PicchiFranchek DrobnicRoberto RonaFrancis Edouard GravierRoberto SaiaFrancisca Alves CardosoRoberto Sanchez-CabreroFrancisca OmorodionRoberto ScendoniFrancisco Alejandro Lagunas-RangelRoberto TeggiFrancisco AlvesRobin HaringFrancisco ArnalichRobin PlaFrancisco EpeldeRobin Singh BhadoriaFrancisco Eugenio Calvo-IglesiasRocco Carmine ServidioFrancisco García-Muro San JoséRocco MeaFrancisco Gonzalez FernandezRocco ServidioFrancisco Guillen-GrimaRocío Llamas-RamosFrancisco J. Flor-MontalvoRocio Martin-ValeroFrancisco J. MedranoRocío Romero-CastilloFrancisco Javier Cano-GarcíaRod MooreFrancisco Javier Fernández CarrascoRodica Ianole-CalinFrancisco Javier González-CañeteRodrigo A. LoiolaFrancisco Javier Jiménez RíosRodrigo MadurgaFrancisco Javier Pérez-RivasRodrigo Martín De San AgustínFrancisco José Fernández-GómezRodrigo OkuboFrancisco Jose Garcia-MoroRodrigo Perez FernandezFrancisco José Gómez GarcíaRodrigo Poblete UmanzorFrancisco MedinaRodrigo Torres-CastroFrancisco MesaRodríguez-Fernández Carmen AntíaFrancisco Miguel Escandell RicoRoel Van OvermeireFrancisco MonteiroRoger E. ThomasFrancisco Nieto EscámezRoger Gutierrez-JuarezFrancisco PerezRohadi Muhammad RosyidiFrancisco SampaioRohan NagareFrancisco-J. Renero-CarrilloRohit GuptaFrancisco-Javier Cantero-SánchezRóisín O'DonovanFranco MusioRoko BjelicaFrancois LefebvreRolf LeferingFrank A. BourgeoisRomain PirracchioFrank JochumRoman KrálikFrank Mayta-TovalinoRoman SmuclerFrank Po Wen LoRoman VavrekFrank Vinholt SchiødtRomano ZannoliFrantišek MurgašRomina-Marina SimaFranziska WeberRomualdas MalinauskasFraser CarsonRonald B. BrownFrédéric BousefsafRonald FisherFrédéric VellaRonald Jan CorbeeFredericus H. J. Van LoonRonald M. IorioFrederik DenormeRondy YuFredrik MolinRong-Kung TsaiFredrik VelanderRongrong LiuFrieder C. KrafftRoni Enten VissokerFrits Van MerodeRosa Alessia BattistaFuad Hamdi A. AbuadasRosa BocciaFu-Hsuan ChenRosa Carla SilvaFulden Ulucan KarnakRosa M. Cárdaba-GarcíaFulvio LauretaniRosa Maria Tapia-HaroFushan TangRosa Meijide FaíldeFuwen HuRosa Roig BerenguerG. Lloyds RajaRosa ScardignoG. SeshikalaRosalie ThackrahGábor OttóffyRosalinda InguantaGabriel D. PeckhamRosario PastorGabriel Dias RodriguesRose Wimbish-CiriloGabriel González-ValeroRoselyn CerutisGabriel RaitaRosemary CaronGabriel StefanRosita BorlimiGabriela Anaya-SaavedraRosnah ShamsudinGabriela De Morais Gouvêa LimaRossella CacciolaGabriela Nérida Condezo CastroRossella RellaGabriele CervinoRossella SgarzaniGabriele GrunigRoswith RothGabriele LombardiRowida MohamedGabriele MascheriniRoxana Adriana StoicaGabriele MelegariRoxana StegaroiuGabriella PunzianoRoy G. BeranGabriella TamburroRubén CalotoGabrielle RussellRubén Cuesta-BarriusoGad SegalRubens Nisie TangoGagandeep SagguRubí Rodríguez-DíazGagliardi Anna RobertaRüdiger PryssGaia Viviana BassaniRudy ArthurGainel UssatayevaRudy PramonoGal TsabanRuei-Nian LiGalatanu Catalin-DanielRuey-Kai SheuGallus BischofRuggero Andrisano RuggieriGalya Georgieva-TsanevaRui DangGang PengRui PoinhosGarasic Mirko DanielRui TorresGarcía-Martínez InmaculadaRui YanGarima DwivediRuida HouGarry KuanRuitu LyuGary StoreyRumen NikolovGąsowska-Bajger BeataRümeyza Turan KazancıoğluGauthaman SukumarRuntang MengGavriil George ArsoniadisRuoliang TangGelei XiaoRuoqi WeiGemma PratRu-Si ChenGengxi LuRussell D'SouzaGeoffrey MadeiraRussell F. D'SouzaGeoffrey Yuet Mun WongRüstem MustafaoğluGeorg ConradsRute SampaioGeorg HalbeisenRute SantosGeorg SingerRuth C. CronkiteGeorge A. KoumantakisRuthmarie Hernández-TorresGeorge JamilRuud G. J. MeulenbroekGeorge JîtcăRuxandra Diana SinescuGeorge ObaidoRuxandra IonescuGeorge PavlidisRyan ChapmanGeorge UmemotoRyan GordonGeorges AdunlinRyan J. WinsteadGeorgescu MadalinaRyan McGrathGeorgi LukovRyan SukGeorgia AngelopoulouRyan T. WhiteGeorgia CasanovaRyland MorgansGeorgina E. MeakinRyo TeraoGeorgios KapousizisRyo YonetsuGeorgios KatsarasRyuichi OhtaGeorgios ManikisS. M. Sohel MahmudGeorgios PapagiannisS. M. Yasir ArafatGeorgios SchoretsanitisS. RamkumarGeovani Lopez-OrtizSaad AlqahtaniGerald AranoffSaad M. BindawasGerald FerrettiSaaima HasninGerald JeromeSaba Ahmed BeighGerald MboowaSabahudin HadžialićGerard LevySabina Antonela AntoniuGerardo FebresSabina Barrios-FernándezGerardo Leyva-GómezSabina KrupaGerardo López MuñozSabina MaraffiGerasimos P. SykiotisSabina ValenteGerdi WeidnerSabrina StranoGerdt SundströmSabyasachi ChatterjeeGerhard LeitnerSachio TakenoGerman Dominguez VíasSachit AnandGermán Gálvez-GarcíaSadeq Al-FayyadhGerman PerezSadia ShakeelGermán PradosSadiq HussainGermina-Alina CosmaSaeid Abbasi-MalekiGerrit GriebSafiya KarimGeu Mendoza CatalánSafwan OmranGiacomo NalliSagar PandeGiacomo PeruzziSahar HoushdaranGiacomo Pietro VigezziSahar ZavarehGiacomo RossettiniSai Pranathi Meda VenkataGiacovelli GiampaoloSajid AliGiada GiorgiSajjad ShiraziGiada TripoliSaju M. D.Giampaolo VettaSakari B. SuominenGiampietro FarronatoSakeenabi BashaGian Luca MarellaSaket A. KottewarGian Luigi NicolosiSalah Al AwaidyGian Maria GrecoSalam Salloum-AsfarGiancarlo CondelloSalil KaripottGiancarlo OttavianoSally AdamsGiandomenico NolloSalma AbedelmalekGianfrancesco FioriniSalman Afroze Azmi MohammedGianluca CiardiSalman GurayaGianluca FeriniSalman ZahidGianluca ManniSalvador Vergara-LópezGianluca SerafiniSalvatore ScolavinoGianpaolo ZammarchiSalvatore VaccaroGibran MancusSam RoweGiedrius BarauskasSamantha HollowayGihan MendisSamantha MaurottiGilberto TadeuSambit MishraGil-ho LeeSameera AbeyrathnaGilles MonifSami AkbulutGiner Alor-HernándezSamia AminGinny LaneSamir Al-AdawiGino SeravalleSamo FokterGioele CastelliSamrad MehrabiGiorgia VaralloSamrita DograGiorgio AiroldiSamson TanGiorgio BoganiSamuel DontaGiorgio GaspariniSamuel José Fonseca MonteiroGiovanna BertiniSamuel Kofi ErskineGiovanna BorrielloSamuele GhezzoGiovanna Maddalena SquintaniSanda Mihaela PopescuGiovanna PerrottiSandeep SharmaGiovanni AudinoSandesh PanthaGiovanni BarassiSandheep SugathanGiovanni BocciaSandip RoyGiovanni CangelosiSandra AlhoyekGiovanni ChiariniSandra BarbalhoGiovanni GalloSandra GavinhaGiovanni MatareseSandra HirschGiovanni MergoniSandra HolleyGiovanni PivaSandra Kalil BussadoriGiovanni RalliSandra PetrauskieneGiovanni RostiSandra PucciarelliGiovanni ScarzelloSandra RojasGirish UpretiSandra Wajchman-ŚwitalskaĢirts BriģisSandrina TeixeiraGisela Margarita Torres AcuñaSandro FeriozziGisela Redondo-SamaSandro Fernandes Da SilvaGisela SantosSandro SerpaGisele Alborghetti NaiSang Ouk WeeGiulia AtzoriSang Tae ChoiGiulia CasuSangamanatha Ankmnal VeerannaGiulia FrissoSang‐Dol KimGiulia LausiSang-Hun JangGiulia Maria MarianiSang-Seok NamGiulia PaganinSanja DabelicGiulia VillaSanjay KumarGiulia ZumboSanjeev KhanagarGiuliano MarchettiSanjiban Sekhar RoyGiulio Di MizioSanthanasabapathy RajasekaranGiulio MariniSanthosh Kumar BalanGiuseppe AndreoniSantiago Martinez-IsasiGiuseppe BattagliaSantiago Navarro LedesmaGiuseppe BorianiSara BernardiGiuseppe ColucciSara CampagnaGiuseppe De PalmaSara CondinoGiuseppe Della PepaSara E. BenhamGiuseppe GagliardiSara El ZahedGiuseppe GulloSara García-BravoGiuseppe IettoSara Huerta-GonzálezGiuseppe La MonacaSara JayousiGiuseppe LanzaSara KhanGiuseppe MaccagnanoSara NunesGiuseppe MandraffinoSara Palomo-DíezGiuseppe MarinoSara SkandraniGiuseppe MasanottiSara Stasik-O’BrienGiuseppe MurdacaSara WettergreenGiuseppe RivaSarah DavenportGiuseppe SignorielloSarah J. Breese McCoyGiusi Antonia TotoSarah MasefieldGiustino VarrassiSarah MyersGıyasettin DemirhanSarah P. MaxwellGizem AratSarah PriorGlauco ChisciSarah RedshawGlayciane Costa GoisSarah RominskiGlenda TinneySarah T. HenesGlenn MelnickSarhang HamaGloria González-MedinaSarmad LatifGloria Jiménez-MarínSasa RajsicGloria Reig-GarciaSashi DebnathGloria SantangeloSaskia HaitjemaGniewko WięckiewiczSathishkumar RamalingamGo AnanSatilmis BilginGodfrey B. WoelkSatish VishwanathaiahGodfrey M. RwegereraSatoru MunakataGonca Gokce Menekse DalverenSatoru TsujiiGonca SoysalSatoshi MurakiGonçalo MarquesSatoshi OsukaGonçalo SantinhaSatoshi YuguchiGonzalo Emiliano Aranda-AbreuSatu KalliolaGonzalo VarelaSaturnino Marco LupiGopakumar GopalakrishnanSatwinder Kaur BainsGopal Nambi NambiSaudade LopesGopi K. PenmetsaSaul FrenkielGoran AugustinSaumil DoshiGoran ErfaniSaurabh JainGoran KrstacicSaurabh RamBihariLal ShrivastavaGöran KurlbergSaval KhanalGordana Kenđel JovanovićSavina SchoenhoferGordon BarbaraSawan JalnapurkarGraciela Caire JuveraSawsan A. ZaitoneGraciela Padilla-CastilloSawsan M. A. AbuhamdahGraham BurgessSawsan ZaitoneGraziano MontaruliSayed Aliul Hasan AbdiGrażyna BączekScot RaabGrażyna BartkowiakScott DrumGrażyna Odrowaz-SypniewskaSeamus O’ReillyGrażyna PutoSean Chun Chang ChenGregor ToporowskiSebastian BlechaGregorio Gonzalez-AlcaideSebastian GłowińskiGregory FirthSebastian ScheurerGregory NeocleousSebastian YuGrigorios G. DimasSebastian ZduńskiGriselda López MaldonadoSebastiano CiccoGrzegorz BlicharzSebastiano FaroGrzegorz DahlkeSebastiano MassaroGrzegorz DrozdowskiSebastiano MercadanteGrzegorz IlewiczSelena FirminGrzegorz JakubiakSelenia Di FronsoGrzegorz KoniecznySeley GharaneiGrzegorz NowickiSema K. AydedeGrzegorz OnikSen-Chi YuGrzegorz ZielińskiSenka Borovac ZekanGrzegorz ŻurekŞenol TuranGuadalupe Molina TorresSenthil AthithanGuangdong TianSeo Young KangGuannan WangSeok Shin TanGuennadi SaikoSeolwoo ParkGuglielmo DurantiSeonaid CleareGuido BruinsmaSeon-Cheol ParkGuido Franco AmorettiSeong Man ParkGuido NotoSeong Tak WooGuido ZavattaSeon-Rye KimGuilherme FurtadoSeoyoon HeoGuillermo Felipe López SánchezSerafí CambrayGuillermo Gómez-DelgadoSerdar SaritasGuillermo HernándezSerena Ann IsaacsGuillermo MoreirasSerena BianchiGuillermo PlazaSerena SalomeGül DikeçSerge BrandGüler Yavuz TemelSergei IgumnovGulzhanat AimagambetovaSergei OsipovGuoxun ChenSergey MoskvinGuru Raghavendra ValicherlaSerghei CovantevGustave MabiamaSerghei SumanGustavo Aviña CerecerSergio A. Mota-RolimGustavo ChristofolettiSergio Calonge-PascualGustavo DesouzartSergio Cinza-SanjurjoGustavo Duarte PimentelSergio Cuevas-CovarrubiasGustavo FernandesSergio Martínez-HuenchullánGustavo MesiasSergio Martinez-VázquezGustavo PimentelSérgio MatosGustavo Rodrigues Makert Dos SantosSergio NavarroGustavo Rodríguez FuentesSérgio Oliveira De PaulaGuzide SenelSerhii LupenkoGwenyth R. WallenSerkan PekçetinGyozo SzolnokySerkan VarolGyula SzaboSeth TarrantH. Cody MeissnerSeunghoo LimHabil Pakai AnnamáriaSeunghyun HwangHadeel HalawehSeung-Hyun MoonHadi JahaniradSevim AhmedovHadiar RahmanSeyed Ahmad Naseri-AlaviHadijah MbwanaSeyed Ahmad Seyed AlinaghiHaewon ByeonSeyed Mahmood Taghavi-ShahriHae-Young KimSeyed Mohammad AyyoubzadehHafiz Muhammad AsifShaaban ElnesrHafiz Tayyab RaufShadab ShahaliHai Canh VuShahabedin RahmatizadehHai DongShah-Hwa ChouHaider QasimShahin Abdollahi FakhimHaidong LuShahin OftadehHaigang WuShahnaz ShahrbanianHaipeng LiuShahram MahmoudiHaishu LinShahrizan JamaludinHaitao LuanShahzadah Nayyar JehanHaitham NobaneeShaimaa A. A. AhmedHakan MaydaShaji KachanathuHalim İşseverShambhu YadavHalis SüleymanShamik ShahHamdi ChtourouShamsher Mohamad Ramadili MohdHamid MoghaddasiShan ZhengHamid OsmanShang-Feng TsaiHamid PakmaneshShankar BakkannavarHammad A. GanatraShankargouda PatilHammad AfzalShannon JarrottHamza El HadiShannon O'ConnorHanaa SalemShanti SandaranHanan A. Hosni MahmoudShaohua WanHanani Abdul MananShaojie XuHang ZengSharada P WastiHani Amir AouissiSharifu UraHani MorganSharmila FagooneeHani N. NaseefSharon DavideskoHani NassarSharon H. MurffHania Rahimi ArdabiliSharon MartinoHanieh MohammadiSharon SmithHanifa BachouSharon StollHanisah KamilahShasha YinHan-Mou TsaiShashwat PathakHanna BandarenkaShatha Ahmed AlduraywishHanna LiberskaShatil ShahriarHanna WetzelShaw-Ji ChenHannah StevensShe Yu LiHannamaria KuusioSheeja Saji VargheseHanqing ZhaoSheila GahaganHans H. StassenSheila Sánchez CastilloHanvedes DaovisanSheila SpadaHanying ChenShekh Md Mahmudul IslamHao MaShelly R. McFarlaneHao XueSheng FuHao YuanSheng ZhuHao-Chin ChangShengkun XieHaoqing WangSheng-Lih YehHao-Yun KaoSheng-Wei LinHaralampos KaranikasSheree YorkHarald MatthesSherif AbdelhamidHaraprasad MondalSherry Yun WangHari S. LuitelShi ChenHarikumar RajaguruShiau Foon TeeHariprasath PrakashShibili NuhmaniHarish ChanderShigeo TakebayashiHarminder SinghShigeo YamamuraHarri H. HakovirtaShihai LiHarshita PatelShih-Chun LeeHarshraj LeuvaShikha JainHarukaze YatsugiShilpa GiteHarun UçakShilpi GiriHasan MujtabaShin Jun ParkHasan S. MirShinji MatsudaHasanain Faisal GhaziShinji TeramotoHashem Salarzadeh JenatabadiShinji YoshiiHashim Talib HashimShinsuke MizutaniHassanah HusinShinta NishiokaHassane HotaitShinya MatsuzakiHatamian-Zarmi AshrafalsadatShinya SatoHatem A. ElshabrawyShinya SuzukiHathal HaddadShinya TanakaHatice AtilganShiow Luan TsayHayder F. N. Al-ShukaShiv VermaHazel MaxwellShiva Hadi EsfahaniHe LiuShivani BansalHe SunShivshankar ThanigaimaniHeather A. DavisShixiong ZhangHeather TilleweinShiyang YanHéctor BurgosShoji OuraHéctor García-LópezShooka MohammadiHéctor González De La TorreShreya PodderHee Young LeeShrilaxmi BagaliHege SletvoldShubha Ranjan DuttaHeidi HillmanShubham PathakHeidi L. DempseyShubhangi ShuklaHeike WieserShuhama RosanaHeimo MüllerShu-I. WuHeinz KleinöderShujiro HayashiHelen AlmondShu-Jui KuoHelen HarmanShujun LiangHelen TrubyShuko NojiriHelena Belchior RochaShumit SahaHelena BiancuzziShunchang MaHelena FelgueirasShuncong WangHelena JoséShung-Te KaoHelena LoureiroShun-Ku LinHelena Sofia Rocha LopesShuo-Tsung ChenHelga MoncayoShuxiang GuoHelge StalsbergShweta KukrejaHélia DiasSibhghatulla ShaikhHelio Junji ShimozakoSiddharth AishwaryaHelmut FrohnhofenSiddharth JaggavarapuHemalatha MunusamySiddharth NathHemant KulkarniSidhhartha SenHendrik BerthSifat MomenHenglong HuSigne TomsoneHenk KoppelaarSilke KuehlHenri AstenSilvia ActisHenri TilgaSilvia CaldeiraHenrik C. BäckerSilvia CataldiHenrique LuisSilvia D’AgostinoHenrique PereiraSilvia D'IppolitoHenrique SilvaSilvia GiannattasioHenry C. Y. HoSilvia GiovanniniHenryk MizerekSilvia GonellaHera AntonopoulouSilvia MonacoHerbert KimuraSilvia Morales ChaineHerbert Ryan MariniSilvia NovíoHeribert Harald FreudenthalerSilvia PerzolliHeriberto Antonio Pineda-EspejelSilvia SoléHero P. WitSilvio TafuriHezekiah FalolaSilviu Dan MandruHidehiro SomekoSima HamadehHidenori KomiyamaSima NaminHidetada FukushimaSimin JamalyHilal OzcebeSimón Yobanny Reyes-LópezHilary Izuchukwu OkagbueSimona Catalina StefanHillary K. KiprutoSimona Lidia SavaHimanshu BatraSimona MirelHipolito NzwaloSimona MitevaHiram CalvoSimona ZaamiHiranya SritartSimone Domenico AsprielloHiroaki KijimaSimone GrassiHiromichi ItoSimone MorselliHiromichi NiikawaSimone VarrasiHironori IshiguchiSimon-Pedro Izcara-PalaciosHiroshi HoriuchiŠimons ŠvirskisHiroshi IrisawaSina HafiziHiroshi KishimotoSina Shaffiee HaghshenasHiroshi KurokiSinan DenizHiroshi YoshidaSinghal RashiHirotaka SawanoSinziana SilișteanuHirotsugu OhkuboSiranjeevi NagarajHiroyuki HanadaSiri PauloHiroyuki WakiguchiSisi ChenHisao NakaiSisitha JayasingheHitomi FujitaSiti Fardaniah Abdul AzizHiu Chuen LokSiti Khuzaimah Ahmad SharoniHo Kee KoonSiti Salasiah MokriHockguan GohSi-Tong ChenHo-Hsuan ChangSitthichok ChaichuleeHolger MuehlanSivakumar NuvvulaHolly GaddSivana Mirella AlibertiHongchang YangSiwei ZhangHong-Duck KimSławomir GawrońskiHongjie JiangSlawomir JarekHongli Sam GohSlawomir TobisHongping JinSławomir WoźniakHongyang XuSlobodanka Lemajic-KomazecHongyi ZhouSneha VivekanandhanHon-Yi ShiSnehil DixitHoraţiu MoldovanSo Jung MunHoria HaragusSofia JohanssonHoria OprisSofia KarachrysafiHoria StancaSohtaro MimasakaHosam AlzahraniSoichiro TakamiyaHossein Hossein-MohandSoledad Pizarro-SánchezHossein ZareSomchai AmornyotinHoufang LiuSomenath ChakrabortyHourei OhSong Toan TranHrvoje BudinčevićSongyi LeeHrvoje KarninčićSongyon ShinHsiang-Ning LukSonia Brito-CostaHsiao-Mei ChenSonia FantoneHsien-Wei TingSonia HornHsin-Chi KoSonia M. OliveiraHsing-Yuan LiuSonia Regina PasianHsuehhsing PanSonia Santander BallestínHua QinSonia ValHuajun LiangSonja J. EllisHuali SuSonja Millert-KalińskaHuang Hui-ZiSoo-Hyun SungHuan-Ji DongSook Fan YapHuaxin ShengSophie Despina VasiliadisHuayi HuangSoraj HongladaromHubert DobrowolskiSorin HostiucHubertus AxerSorin Radu FleacaHucheng WangSotirios FortisHuey-Hong HsiehSotirios KakavasHugh GashSoumitra DasHugo CordeiroSoyar SariHugo Leiria NevesSravan PerlaHugo Valadares SiqueiraSrdjan MarkovicHui SunSree Lekshmi Sreekumaran NairHuiling ChenSreekanth Kumar MallineniHuitzilihuitl Saucedo-OrozcoSreevatsan RaghavanHuizhou YangSreyashree BoseHulisani MatakanyeSrijani BasuHumberto HernandezSrinivas PittalaHumberto Javier Rodríguez HernándezStacey SloneHung-Chang LiaoStamatoula TsikrikaHung-Pin TuStanislav KotlyarovHunter J. BennettStanislav PekarskiyHuriviades Calderón-GómezStanisław HorakHurriyet YilmazStanislaw OłdziejHusin AlatasStasja DraismaHyang Yuol LeeStavros DimopoulosHye Chang RhimStavros PitoglouHyejin JeonStefan AmbsHyeong-Tak LeeStefan Cristian VesaHyo Nam SongStefan HadzicHyo Sun JungStefan HertlingHyo-Jung JeongStefan Mandić-RajčevićHyoung Chul ShinStefan Marian FrentHyoung-Kil KangStefan NaydenovHyung Rok WooStefan OliverHyungoo KangStefan ReuterHyung-Seok KimStefan SajdakHyun-Jeong BanStefania CantoreHyunjin KimStefania CerinoHyunjung KimStefania CimbanassiI. Chien WuStefania ManconeI. Gusti Ngurah Edi PutraStefania MarsiliI. Lin WangStefania OrrùIain A. BrownleeStefania ToselliIan HodgsonStefania TroiseIbo H. SouwerStefania ZoiaIbolya FülöpStefanie L. RussellIbraheem M. KarayeStefano BocaIbrahim Ahmed ShaikhStefano D'ErricoIda AringerStefano LafranceschinaIda Laudanska-KrzeminskaStefano LasaponaraIdris ArslanStefano PaganoIdris LongStefano RestainoIeva Meidute-KavaliauskieneStefano RizzaIevgen ZaitsevŠtefica MikšićIgnacio Ramos-VidalSteinn SteingrimssonIgnacio RefoyoStella ChristopoulouIgnacio RintoulSten HellströmIgnacio TartaraStepan FeduniwIgnazio CondelloStephan HeisingerI-Hsuan LinStephan LauxmannIina SavolainenStéphane SanchezIkkei TamadaStephanie HunterIkram SghaierStephanie Rodríguez BesteiroIlaria CataldoStephen BagwellIlaria PinaStephen D. EdwardsIlaria Tocco TussardiStephen E. ForemanIldiko RaczStephen IbidunniIleana Heredia-PiStephen WechslerIlia StamblerStephnae BilodeauIlias MahmudSteve Simpson-YapIlke Coskun BenlidayiSteven Baumruckerİlknur KaleliSteven G. Kovenİlknur Kıvanç AltunaySteven SilversteinIlsang KoSteven WallerIl-Youp KwakStijn KeereweerIm Hee ShinStuart RundleImelda S. WongStylianos E. TrevlakisImena RexhepiStylianos MystakidisImtiyaz Ahmad WaniStylianos TomarasInaki Pastor-PonsSu H. WangIndrajit ChowdhurySubhash BhallaInés Aguinaga-OntosoSubhashis DasInes Bilic-ĆurčićSubin Thenkunnel SurendranInés Casado-VerdejoSubrat BhattamisraInês FranciscoSubrata DebInes KožuhSuchada KongkiatkamonInés Llamas-RamosSudeep TanwarInes VillanoSudha BhavanamInge Hansen BruunSudipta BiswasIngrid PrkačinSufaru Irina GeorgetaInmaculada Mateo RodríguezSuhail Sarwar SiddiquiInna FeldmanSuhaily Mohd HaironIn-Seok SongSujata ChoudhuryIoana Dana AlexaSujit Kumar DeIoanna ChatzigiannidouSukru Serdar BalciIoannis AnagnostopoulosSuleman AtiqueIoannis AnyfantisSuleman LazarusIoannis GeorgakopoulosSuliman MohammedIoannis IntzesSulistyawati SulistyawatiIoannis KoutelekosSultan Ayesh Mohammed SaghirIoannis LiampasSumaiya ThaseenIon PopaSuman ChaudharyIonescu Tiberiu-ConstantinSumio AkifusaIonut DonoiuSun ImIonut LuchianSun Mi ShinIonut SchiopuSun-Ae KimIosune Salinas-BuenoSunčica RocaIppolito NotarnicolaSuneel KumarIram FatimaSung Hee ParkIrena IlicSung Ho MaengIrena MilaniakSung ParkIrena MladenovaSunghoon ShinIrene Carrillo MurciaSung-Hyoun ChoIrene Cortés-PérezSung-Soo KimIrene Lizano-DíezSunil RaiIrene Sandoval HernándezSunil S. KarhadkarIrfan KhanSunita KeshariIrfan MohamadSunitha ZechariahIrfan UllahSuparat PhuanukoonnonIrfanuzzaman KhanSuparerk JanjarasjittIrina GradinaruSuparshya Babu SukhavasiIrina KiselevaSurender SinghIrina MocanuSurendiran BalasubramanianIris ManorSuresh SagadevanIris UdasinSurya KantIrmina MorawskaSusan A. LetvakIrniza RasdiSusan McClementIrshad AhmadSusan WakenshawIsaac López-LavalSusana HenriquesIsaac Machorro-CanoSusana Navalpotro-PascualIsaac RheeSusana Sofia MiguelIsabel ColmeneroSusanna SummaIsabel Corrales GutiérrezSusanne RospleszczIsabel De Jesus OliveiraSushanta Kumar MishraIsabel Escobio-PrietoSushil MiddhaIsabel FernandesSushma DahalIsabel María Fernández-MedinaSusie YoonIsabel Poiares BaptistaSusumu ItoIsabel Rodrigo MartínSusumu SawadaIsabel SalvatSuvasini LakshmananIsabelle GermainSuveenkrishna PothuruIsao IshiiSuzan CinarIshita BasuSuzana OtaševićIsilda RibeiroSuzanne HanserIslam MohamedSuzanne O’NealIsrael Casado-HernándezSven DreyerIssam TanoubiSvenja Dannewitz ProssedaIssei ShinoharaSvenn-Erik MamelundIstván TarrósySvetlana KrasnovaItay BarneaSyafrizal Helmi SitumorangItzhak OrionSyed Arman RabbaniItziar Sobrino-GarciaSyed Hassan RazaIulia Dana PopescuSyed MahmoodIuliia PavlovaSyed Mohammed BasheeruddinIva SpeckSyed QadriIván CarreraSyed Thouheed AhmedIván Chulvi-MedranoSyed-Abdul ShabbirIvan ĆukSylvain BrousseauIván EcheverriaSylvère StörmannIván Herrera-PecoSylvester Reuben OkekeIvan MiskulinSylvester Yao LokpoIvan PiresSylvia MignonIvan ŠklebarSylwia Katarzyna KałuckaIvan ŠošaSylwia ŁaganIvana DjuricicSylwia Zakowska-BiemansIvana HromatkoSze ChanIvana TadicSzymon Łukasz DraganIveta VrabkovaSzymon SkoczyńskiIvica MatićTadashi ItoIvica PelivanTadashi NakamuraIvo Dumic-CuleTadeusz KufelIwao UeharaTaha MehanyIwona Olszewska-CzyżTahir AnsariIwona Paradowska -StankiewiczTahseen Al-wattarIwona StaniecTaiwo AremuIwona ŚwiątkiewiczTaiyong LiIzabela DudaTakahiro KannoIzabela ZakrockaTakamasa MiyauchiIzabell CraciunescuTakanobu HirosawaIzabella LeckaTakashi AriieIzabella UchmanowiczTakashi MatsushimaIzidor MlakarTakashi NomizoIzolde BouloukakiTakayuki KoikeJ. Andrew OnesimuTakehiro MichikawaJ. Benito Bouza-RodriguezTakeshi MiyamaJ. J. Sylvia IVTakeshi UnokiJ. JumadiTakeya OnoJ. Scott LeeTakuji TanakaJacek BucznyTakuma InagawaJacek MatysTakuya OmuraJacek WilczyńskiTalha Mahboob AlamJack ThepsourinthoneTamar BasishviliJacob E. TeitelbaumTamara BackhouseJacopo GiamelloTamara GajićJacopo LanzoneTamara GoldsbyJacopo PavanTamara GuénounJacopo TalluriTamara Nikolic TurnicJad Adrian WashifTamara Pawlaczyk-KamienskaJae Wook YangTânia CorreiaJae Yong YooTanja J. MiličićJaehee LeeTanuj WadhiJaeho ChungTanya HeynsJae-seop OhTao PengJaeson CallaTao TongJae-Sung YooTariq A. ShahJagadheshwar BalanTariq Alwada’NJagdeep KaurTaru SinghJagdeep RahulTaryn KlarnerJagdish KhubchandaniTasuku OkuiJahanpour AlipourTatiana ChristidesJaideep MahendraTatiana De Oliveira SatoJaime Martinez-CastilloTatjana GoranovičJaimie Z. ShingTatsuo KandaJakkrawut MaitipTatsuo NakamotoJakub DuszczykTatsuya FujikawaJakub KokštejnTatsuya NishinoJakub KołodziejczykTaylor L. WattersonJakub KufelTe-Chih WongJames A. KingTe-Chun ShenJames C. OlesonTed McdonaldJames Chung Wai CheungTeddy Weifa YangJames FrideresTe-Feng YehJames PowersTeija RantalaJames R. S. HockerTejeshwar RaoJames W. DearingTelung PanJames W. McKowenTerence LoveJames W. SonneTeresa FarroniJames WalterTeresa Letra MateusJames WilliamsTeresa PerraJamie SmithTeresa Pozo-RicoJamie TaylorTeresa Rubio-TomásJan EggerTeresa Sanclemente-HernándezJan Irma PoelaertTeresa Silveira LopesJán LackoTeresita Paz-MaldonadoJan LexellTerézia RončákováJan MuzikTerry Jacob MathewJan PithaTerry YoungJan SchröderTeruhiko ImamuraJan UlfbergTeruya KomatsuJanani VaradarajanTeruyoshi AmagaiJane AkisterTetsuo OhyamaJane K. NguyenTetsuya HirataJane KuepferThammanard CharernboonJane M. GannonThashmee KarunaratneJane McElroyTheano KokkinakiJanet W. M. EllisTheerut LuangmonkongJangPyo BaeThelma SpindolaJanice BakerTheng Choon OoiJanice R. HermannTheodor TirilomisJanine IdziakTheodore WeinJanine WhiteTheodoros FouskasJanna G. KoppeTheodoros GiovazoliasJanusz OpolskiTheofanis ChatzistamatiouJanusz SielskiTheresa WestgårdJanusz SieroslawskiThersilla OberbarnscheidtJaroslava Paulasova SchwabovaThibault CanceillJarosław Jaszczur-NowickiThien-An LeJarosław PinkasThierry Oscar EdohJasenka Gajdoš KljusurićThiha Tin KyawJasenka WagnerThijs T. WingelaarJasmina IvaniševićThilo SpeckmannJasna KaračićThomas KallarakkalJason BrumittThomas KrauseJason FlattThomas PerreaultJason J. WangThomas Pierre LecompteJason James TurnerThomas SchubertJason S. BuhrmanThomas T. H. WanJason T. TzenThomas WaldhoerJasper Van AsscheThomas WanJavad Anjom-ShoaeThomas WenzelJavad Javan-NoughabiThuane RozaJaveed ShaikThyago C. C. NepomucenoJavier Almela-BaezaTian KangJavier CarniceroTian LiJavier Gil-GimenoTianjun WangJavier Marco-LledóTibor BruntJavier Martín NúñezTifeng JiaoJavier Ortuño SierraTiffany Champagne-LangabeerJavier Sanchis ZozayaTiffany J. Ríos-FullerJavier TarangoTiffany JonesJaya Shanker TedlaTiffany WashingtonJayabrata BhaduriTihomir MustakovJayanta MondalTihomir VasilevJazmín Mora-RíosTiija RintaJean Claude De MauroyTijana BojićJean De La RosetteTilakavati KarupaiahJean LimongiTill BraunschweigJean RossTim HulsenJean TchuencheTim SandleJean VillerdTimothy MakubuyaJean-François SubraTin ČohadžićJean-Gabriel ContaminTina TomazicJeanine P. AbronsTinakon WongpakaranJeanne SlatteryTing Kin NgJean-Paul CalbimonteTing WangJee-Hyun HwangTinghao ChenJeena GuptaTing-Lin YenJefferson AraujoTingting ZhangJefferson Romáryo Duarte Da LuzTingwei ChangJeffery HeilesonTino PrellJeffrey E. JanisTiziana CiarambinoJeffrey J. HuangTiziana GreggiJeffrey Pradeep RajTiziana QuartoJeffrey S. ForsseTobias Kvist StrippJeffrey SilberzweigTobias RufJehan ZebTobias WeinmannJelena MaksimovićTom TreasureJelena MusulinTomaso VecchiJelena S. ĆirićTomasz BogielJelica Grujić-MilanovićTomasz CudejkoJenna SciscoTomasz FuchsJennifer A. CrittendenTomasz GabryśJennifer BrayTomasz GigolJennifer ChopraTomasz GołębiowskiJennifer Creese (Ireland)Tomasz KuligowskiJennifer Creese (UK)Tomasz MichalskiJennifer F. MoffatTomasz MorońJennifer FredianiTomasz PobożyJennifer LiraTomasz PodgórskiJennifer Miner WeaverTomasz PorażkoJennifer MytychTomasz RakowskiJennifer PascaliTomasz RechbergerJennifer SalinasTomasz SobierajskiJennifer SneddonTomasz SzymborskiJennifer ZitserTomasz UrbanowiczJenn-Ming YangTomasz WolnyJenny StenbergTomaž GoslarJenny Vilchis-GilTomislav RukavinaJenny WilkinsonTommasina PianeseJeoung Mi KimTommaso ScquizzatoJere KoskelaTomohiro UdagawaJeremy D. P. BlandTomoki MaedaJerilynn C. PriorTomoki SekiguchiJernej DolinsekTomoko ItoJerome CleofasTomomitsu MiyagakiJerónimo González-BernalTomoya ItataniJerris HedgesTomoya NishinoJerzy ChudekTomoyuki FujiokaJerzy WilińskiTomoyuki KanazawaJesper Møller JensenTomoyuki KobayashiJessica HillTonghun HwangJessica Rial-VázquezTongtong LuJessica StubbingTony SkapetisJesus Enrique GarcíaTope OyeladeJesús Jiménez-LópezTorbjörn LedinJesús María López-ArrietaTorfinn HynnekleivJesús Martínez De La CalToshiaki AraJesús Molina-MulaToshiharu MitsuhashiJesús Rivera-NavarroToshihiro KawaeJesus S. JimenezToshinobu TakedaJesús-Nicasio García-SánchezTosin PopoolaJeyasakthy SaniasiayaTracey HodurskiJi LiuTracey L. WoodliefJi Yeon ChoiTracy ComansJiabin YuTran NguyenJiacun WangTrevor ArcherJia-Feng ChangTrevor BraselJia-Hao ChangTrisha L. AmboreeJiahe LiTsehay Admassu AssegieJian LiTsige-Yohannes HabteJianfei SunTsukasa YoshidaJianhong WangTsu-Ming YehJian-Hong YeTsung-Cheng HsiehJianing FuTsuyoshi TajikaJianwei ZhengTudor Sorin PopJianxiang XueTuire KuusiJiao YuTullio PiardiJiawei LiuTuo GaoJiayue GuoTuo MengJie ChenTurgut KarakoseJi-Eun ShinTusneem KausarJihfu TuTyler Marie KilesJihui ZhangTzu-Chueh WangJijia WangTzu-Hua WuJill MorganTzu-Yen HuangJi-Man KimUbaldo BonuccelliJin JunUla MahadevaJin WangUldis RubinsJina OhUlf TitzeJin-Ding LinUlises De La Cruz-MossoJingan LiUlrich KellnerJingyu WangUlrich SchmochJinpitcha MamomUmberto GaragiolaJinsong HaoUnai AzcarateJinwei XieUrna KansakarJiong ZhouÚrsula FauraJirada SringeanUsharani ChelladuraiJiro OkumuraV. Krishna KumarJisun LeeVaclav BuncJitendra KanshanaVáclav HönigJitendra SinghVadim DukhaninJitka VeldemaVahid RakhshanJi-Won RyuVaios PeritogiannisJiwon ShimVal LivingstonJi-Yeon KimValentina Brzović RajicJo KatoValentina ChkoniyaJoachim VossValentina Oana BudaJoan Francesc Fondevila GascónValentine Joseph OwanJoan Sole-PlaValeria Soto-MendozaJoan VicianoValeria VendittiJoana CimaValeria ViscoJoana Sofia Dias Pereira De SousaValeriana GuijoJoana SousaValeriia HaberlandJoanna BagińskaValerio De StefanoJoanna BaranValerio GiacconeJoanna BogusławskaValerio PellegriniJoanna ChylińskaValliappan RamanJoanna Dec-PietrowskaVanaja Krishna NaikJoanna Janiszewska-OlszowskaVance G. NielsenJoanna KossewskaVan-Dai TaJoanna LukasikVandana VarmaJoanna Popiolek-KaliszVanesa Cantón-HabasJoanna Rosak-SzyrockaVanesa ZorrillaJoanna Smoluk-SikorskaVanessa CamilleriJoanna SobiakVartika TomarJoanna WitkosVasco PoncianoJoanna Wnęk-GozdekVasile BaltacJoanne PikeVasileios MemosJoannes ChliaoutakisVasilevskaya EkaterinaJoão Carlos MacedoVasiliki TsigkouJoão De Melo NetoVasilios PergialiotisJoão Gama MarquesVassiliki BoumbaJoão M. C. TeixeiraVassilios PanoutsakopoulosJoao MamedeVassilis MakrakisJoão Manoel Silva JúniorVasyl MartsenyukJoão Manuel Garcia Do Nascimento GravetoVeerabhadrappa S. T.João Paulo Mandes TribstVeerle GarrelsJoão Pedro BaptistaVenkatakrishnan RengarajanJoão Rafael AlmeidaVenkateshwari VaradharajanJoaquim ChalerVera LomazziJoaquim Murray Bustorff-SilvaVera StaraJobst LandgrebeVeronica Perez-De La CruzJoel LexchinVerónica Velasco-GonzálezJoeri J. PenVeronica VernonJohan BorgVeronika AleksandrovychJohanna EspinViann Nguyen-FengJohanna Järvinen-TassopoulosVibhu Krishnan ViswanathanJohanna Lisa DegenVicente GabardaJohn A. CaldwellVictor Diogho Heuer De CarvalhoJohn A. TayekVictor Doménech GarcíaJohn Amson CapitmanVictor TamboneJohn B. KortbeekVictor TiberiusJohn F. NewmanVictor-Alexandru BriciuJohn J. HalperinVictoria GonzalezJohn Jairo AraujoVictoria Mazoteras-PardoJohn McNuttVictoria Valeriivna RodinkovaJohn RiceVictor-Vlad CostanJohn VakrosVida DemarinJohn Wayne FisherVidar HjellvikJolanta KisielewskaVidya KudvaJolanta Klukowska-RötzlerViet TranJolanta MackowiczVijay IndukuriJolanta MasiakVijay Kumar VermaJonathan BayuoVijay PandeyJonathan D. KarpVijay SivaramanJonathan LelandVijaya Anand ArumugamJonathan TownendVijaya Saradhi MettuJonathan WislerViji VijayanJong Cheol ShinVikash AgrawalJong-Chul YangVikash KansalJongeun LeeVikram MittalJonghwa JangVikrant RaiJonna OjalammiViktar LemiasheuskiJoonhong ParkViktor ChrobokJorddy Neves CruzViktor FredrichJordi Colomer FeliuViktoriia VovkJordi Franch-ParellaViktoriya PantyleyJorge Álvarez-CervantesVilma PinchiJorge Amate-RomeraVimala NunavathJorge Calvillo-ArbizuVimala RamooJorge CervantesVinay GoyalJorge Góngora-RodríguezVinayak VijayanJorge HochstetterVincent Tsz Fai ChowJorge Limon-RomeroVincentas VeikutisJorge Lopes Cavalcante NetoVincente Javier Clemente-SuarezJorge Luis CandiottiVincenzo De RosaJorge Luis García-AlcarazVincenzo MalagninoJorge Ma NavarroVincenzo MarcotrigianoJorge Murillo-GonzálezVincenzo ZanonJorge N. R. MartinsVineet Kumar KamalJorge OlivaresVineet MittalJorge Rodolfo BeingoleaViola VambolJorge Rojo RamosVioleta DediuJorge UmbelinoVirag VasJorge Velázquez SaornilVirginia Alcaraz- RodríguezJoris HeyseVirginia Sánchez-MonroyJosé A. CernudaVirginia Serrano-GómezJosé A. F. CostaVishakha Anand PawarJose Angel FontenlaVishal ChavdaJosé Antonio Marín MarínVishnunarayan Girishan PrabhuJosé Antonio MingoranceVishnuprabu Durairaj PandianJose Antonio NaviaVita JagričJosé Antonio Ponce-BlandónVitaly PostoevJosé Bañuls-RocaVitaly SchetininJosé Bruno Nunes SilvaVitor ParolaJose C. AdsuarVítor RaposoJosé Carlos Tavares CarvalhoVitor RodriguesJosé D. UrchagaVittorio AprileJose E. Lozano-RizkVittorio FineschiJosé Eduardo TeixeiraVivek P ChavdaJosé F. Gómez-ClavelVivek SinghJose Fernández-SáezVivian VimarlundJosé Francisco Rodríguez-VázquezViviana AndreescuJose Granero-MolinaViviana IvanJose Gutierrez-NaranjoViviana OnofreiJose Jonatan Cortés-MartínVlad RadoiasJosé Juan Carrión-MartínezVlad TicaJose L. García-SoidánVladimir DodevskiJose L. Gómez-UrquizaVladimir PorochJose LlosaVlad-Olimpiu ButiurcaJosé López-CastroVlaho BrailoJosé Luis Martín-ContyVlasios SotirchosJosé Luis Maté MuñozVojko MatkoJosé Luis Pérez-CastrillónVolkan YilmazJosé Luis Romero-BejarVolker GehrauJosé Luis Sánchez-SánchezVsevolod V. KonstantinovJose Luis UbagoWafa GhardallouJose M. GamonalesWagih Mommtaz GhnnamJosé M. Garrido-MolinaWai Sum Amy LeeJose M. Rodriguez FerrerWakiro SatoJosé Manuel Ferreira MachadoWally WojciechowskiJose Manuel LopesWalter E. KaufmannJosé Manuel López GarciaWalter F. RiesenJosé Manuel MendesWalter J. FlapperJosé Manuel Ortiz-MarcosWalter L. StrohmaierJose Manuel Santos-JaenWalter R. SchummJosé Manuel Tomás-MiguelWalter SchummJose María León-RubioWanyu XiJosé María López GullónWarawut ChaiwongJosé MendesWasan KatipJosé Miguel Seguí-RipollWasiq KhanJosé Niño-MoraWayne CarterJosé Pedro MartinhoWei ChengJosé Ribeiro-GonçalvesWei HuaJosé Rubén Herrera-AtocheWei WuJose Vicente-SamperWei XingJose ZafraWei XueJosefa Gonzalez-SantosWei YanJosefine AtzendorfWei ZhouJosep Carles Benítez-MartínezWeixin LiJoseph BaumgardenWeiyan YinJoseph Calvin KouokamWelles Antonio Martinez MorgadoJoseph J. CrivelliWells UtembeJoseph MukuniWenbing ZhaoJoseph NassifWen-Chen WangJoseph R. El-KhouryWenchen WuJosephine StorekWen-Cheng LaiJosé-Víctor RodríguezWendy BonythonJosh SeimWendy Lynne GilleardJoshua E. MuscatWendy OakesJoshua SamsoondarWenfei ZhuJosie HenleyWenfeng ZhengJosip DelmisWeng Marc LimJosip TomićWeng-Kun LiuJosipa BukicWen-Hsu LinJosipa KernWenhui ZengJosko BozicWenpan LiJosue MartosWenzhen CaoJothi VargheseWerner CordierJovan GardasevicWhitney A. BullockJovana KojićWidaad ZamanJovana TodorovicWiesław DeptuŁaJowy Yi Hoong SeahWiesław FideckiJoyce ChungWilbert LawJoyce StechmillerWilfredo De Jesús-RojasJoydeep ChakrabortyWilliam B. GrantJoydev GhoshWilliam D. RhineJuan Aníbal González-RiveraWilliam HammondJuan Antonio Roche CárcelWilliam JankowiakJuan Antonio Valera-CaleroWim CalameJuan C. Guevara-PérezWioletta MędrzyckaJuan Carlos De La Cruz-MárquezWi-Young SoJuan De Dios Benítez SilleroWłodzimierz KołodziejczakJuan González-PascualWojciech EliaszJuan Jesús García-IglesiasWojciech KiebzakJuan Jesús Gutiérrez CastilloWojciech PaslawskiJuan José Criado-AlvarezWojciech StykJuan LaboraWojciech WojtkowskiJuan LagoWojciech WolanśkiJuan López BarreiroWolfgang LeisterJuan Luis Delgado-GallegosWolfgang SchoppekJuan Luis RubioWon Hee LeeJuan Luis Sánchez GonzálezWon-Bae ParkJuan Manuel Muriel-SánchezWoochun JunJuan Miguel Martínez-GalianoWoohyun YooJuan OyanedelWoojoo LeeJuan Pedro MartínezWoorim KimJuan Pedro Martínez RamónWoraphon YamakaJuan R. CocaWouter KokJuan Rodríguez MansillaWu WangJuan Valdés-StauberWuqiang ZhuJuan-Francisco Álvarez-HerreroWycliffe W.Simiyu NjororaiJuan-Francisco Peña-CardellesXavier X. Sastre-GarauJudith M. ScottXi LouJudith Sánchez-RayaXia HaoJudy GopalXiang WangJudyta KabusXiang WuJulian KenyonXiang ZhaoJulian MangesiusXiangli ZhaoJulián Solís García Del PozoXianglong ZengJuliana FernandesXiangping ChuJuliana Melo Teruel Biagi CamargoXianhua LiuJulie DeMartiniXiaodong ZhangJulie Dextras-GauthierXiaohe XuJulie G. LedfordXiaojie ChuJulie ScorahXiaojie SunJulie SwartzendruberXiaojun DingJulie VaiopoulouXiaojun LuJulie WalabyekiXiaojun ShanJulien AncelXiaokang WuJulien CabatonXiaoming LiJulio César Almanza-PérezXiaoqin WangJulio Plaza DíazXiaoqing LiuJulita KulbackaXiaoting QinJumpei SaitoXiaowen TianJun KohyamaXiaoyan WangJun QiXiaoyu LanJun YasudaXiaoyuan WangJun YoshinagaXin QiJunaid AhmedXin ZhongJunaid RashidXinan WangJunaidi KhotibXinli DuJune L. GinXinyu FangJung Hwa LimXiucai YeJung SunwooXosé López-GarcíaJung-Ah ChoiXuanyi NieJung-Pin LaiXuebing CaoJung-Youn ParkXulong ZhangJunhyung KimXuqun You‪Junia Nogueira De BritoYa O. MezhuevJunmei WangYaara Endevelt-ShapiraJunrui ChengYael ArbellJunxiang ChenYafeng LiJunya KusumotoYahia Z. HamadaJuraj NemecYa-huei WangJurema Corrêa Da MotaYajuan SuJürgen GermannYamakawa MiyaeJurica FerenčinaYan GuoJusak JusakYanan LuoJustin LernerYanfang ZhaoJustus MarquetandYanhui GuoJustyna Berniak-WoźnyYanis Toledano-MagañaJustyna E. GołȩbiewskaYaniv EfratiJustyna Kosydar-BochenekYanjie ZhangJustyna KujawskaYao SongJustyna Opydo-SzymaczekYary VolpeJustyna Podgórska-BednarzYashbir SinghJustyna RójYasir WaheedJustyna SikoraYassmin MoatasimJustyna WojtaśYasutake TomataJuzer ShabbirYasuyuki TokinagaJyh-Jeng WuYawei LiK. C. SantoshYazed AlsowaidaK. G. PrasanthYazed Saleh AlsowaidaKa KimYazeed Yasin GhadiKadri SuijaYazhen YangKah Hui WongYekbun AdiguzelKa-Huen YipYen-Ching ChuangKai Guo (China)Yen-Ming HuangKai Guo (USA)Yen-Nung LinKai Zhen YapYeong Jun JuKaia LaidraYeon-Ha KimKaidong WangYesu AddepalliKaila L. StipancicYi HuKaiway LiYi LiangKajetan SucheckiYi YangKalaimani ElangoYi ZhenKalliopi Anna PouliaYi-Chih LeeKalliopi MegariYijia ZhangKálmán ImreYijun PanKalpana NathanYimei JiaKalthoum TizaouiYiming JiKalyan Sundar GhoshYiming TangKamal Kant SahuYin Ling WooKamal ShahYinfeng ZhangKamalesh ChakravartyYing-Chieh LiuKamil BarańskiYingming YangKamil KoszelaYingying ZhuKamil M. FramYin-Hwa ShihKamil Reza KhondakarYin-Lin WangKamil ZaworskiYi-Wen ChiuKamila KorzekwaYmkje Anna De VriesKamila Pokorska-NiewiadaYogi Tri PrasetyoKamila Ziółkowska-WeissYohan KerbageKam-Ming MokYohei SawayaKamran AshrafYohei TomiokaKamran HaratiYoichiro OdaKan LiYokrat TonKanako NoritakeYolanda Castellote-CaballeroKanokporn PinyopornpanishYolanda GarcíaKapo WongYong GuKara GavinYong ZhuKaranovic DanijelaYongjun RenKarel KostevYongkui LiKarel Van Den BoschYongtao LuKaren HardeeYoon Jeong ChoKaren KierYoshihisa HirakawaKaren L. SternYoshikazu Honda-OkuboKaren M. KedrowskiYoshimasa MiuraKarice K. HyunYoshinobu OdakaKarin BammannYoshinori MoriyamaKarin ConinxYoshiro HoraiKarl HessYoshiro SaitoKarl HusaYosra A. HelmyKarl Kai McKinstryYosuke MatsuuraKarl Uwe PetersenYoufeng ZhuKarl WegscheiderYoung Kyun KimKarl-Arne JohannessenYoung-Chang P. AraiKarl-Heinz LadwigYoung-Joo AhnKarol DąbrowskiYoungjun ChoiKarol KonaszewskiYoung-Sang KimKarol KrólYoungsub OhKarol PilisYu AkaishiKarol RycerzYu TakahashiKarol SzylukYu’E LiuKarolina ChilickaYuan MengKarolina HoffmannYuan-Chuan ChenKarolina KossakowskaYuane JiaKarolina SobczykYuanmay ChangKarolina SzulcYuanwei XuKarthik H. ShankarYubbu PutriKarthik RamamurthyYu-Chao ChangKarthik RamasamyYu-Chi LeeKarzan IsmaelYue ZhangKasi VegesanaYueh-Juen HwuKasper SipowiczYuemin BianKata CsekoYueqi YanKatarina SebekovaYuezhou ZhangKatarzyna DomaszewskaYufei CuiKatarzyna HojanYugo IwayaKatarzyna HolcmanYuh HasegawaKatarzyna Kajak-SiemaszkoYuhei MatsudaKatarzyna LachowiczYuhki SatoKatarzyna LewtakYuichi MurakiKatarzyna ŁokiećYuji KabasawaKatarzyna M. TycYuji ShimizuKatarzyna Mańka-MalaraYujie SuKatarzyna Mocny-PachońskaYukihiro WadaKatarzyna Ogrodzka-CiechanowiczYukio TsugihashiKatarzyna OszajcaYulia O. BobrovaKatarzyna Plagens-RotmanYuliana Jiménez-GaonaKatarzyna RolfYuma YokoiKatarzyna SygitYu-Min ChoKatarzyna SzewczykYun HanKatarzyna TomaszekYun WangKatarzyna Zaborowska-SapetaYun ZhuKate CheneyYung YauKate FrazerYung-Ching WengKate HsuYung-Sheng ChenKaterina AntoniouYung-Shuo KaoKaterina KatsaniYunis M. MayasiKatharina BeyerYunxiao ZhangKatharina DiehlYun-Yi ChenKatharine W. FernstromYuriy L. Vasil'evKatherine E. StraffordYuriy Vasil'evKatherine N. ThekenYu-Sheng ShenKatherine S. SalamonYusuke EndoKathiravan SrinivasanYusuke KajiwaraKathleen LemanekYusuke KatayamaKathleen M. SarberYusuke OsawaKathrine Jáuregui-RenaudYutaka OwariKathryn JarvisYu-Wei FangKatia PinelloYuzuru SakamotoKato HideoYves ReingewirtzKatrin ScheinemannYvonne LanganKatrina HinsonYvonne SchafflerKatrina O'HalloranZacharias PapadakisKatsuki TsuchiyamaZaffar Ahmed ShaikhKatsuo OshimaZahra AshkavandKaviraja UdupaZahra BagheriKavita BatraZahra GharibiKavita MathurZahra PanjaliKawoun SeoZakariya AlgamalKawsar AhmedŻaneta Kimber-TrojnarKaya WitteZara SteinmeyerKazuaki ImaiZbigniew AdamiakKazuhiko KibayashiZbysław DobrowolskiKazuhiko KotaniZeinab GhazanfariKazuhiro MiyataZengliang RuanKazuhiro P. IzawaZenon PogorelićKazuki HiraoZhao YangKazuki TokumasuZhen QiuKazunori NagasakaZhen ZhouKazuomi SatoZheng Feei MaKeegan GuidolinZheng LiKeeren Sundara RajooZhenglin GuKeisuke FujiiZhenwei YuKeith RochfortZhenxuan ZhangKeith SteinhurstZhenyuan WangKelly De JesusZhenzhen ZhengKelvin Ian AfrashtehfarZhi YangKemal GörürZhiping LiuKenichi YamadaZhonggang FengKenioua MouloudZhongwei HuangKenji HayashidaZhongwei ZhangKenji ImaiZiaul Hasan RanaKenneth C. LandZifei LiangKenneth R. FosterZihao OuKenny Yat Hong KwanZiheng ShangguanKensaku MatsunamiZikai HaoKensaku TakaseZlatko KljajicKensuke KumeZo RamamonjiariveloKensuke UraguchiZoard T. KrasznaiKentaro MatsuiZoe DaveyKeren Cohen-HagaiZohreh MousaviKeren DopeltZoka MilanKersti Alice YlloZoltan HeroldKeshrie NaidooZongshuan DuanKeumseok KohZorica JanjetovicKeun Ho RyuZorica Terzic-SupicKeun-Yeong JeongZoubir DjeradaKevin B. KnopfZsolt FeketeKe-Vin ChangZsuzsanna SzaboKevin FujiZubia VeqarKevin MeestersZulqurnain SabirKevin P. UribeZunwei ChenKevin T. FujiZuyi FangKevork PeltekianZuzana ŠoltysováKeya SenZuzana ŠpacírováKeyla Vargas-RománZvonko Rumboldt


